# The role of soot oxidation on the optimization of chitosan/soot aerogel beads for heavy metals and dyes adsorption

**DOI:** 10.1039/d6ra09330b

**Published:** 2026-11-02

**Authors:** Shehryar Ahmad, Konstantinos N. Maroulas, Mariano Sirignano, Martina Salzano de Luna, George Z. Kyzas

**Affiliations:** a Department of Chemical, Materials, and Production Engineering (INSTM Consortium-UdR Naples), University of Naples Federico II P.le Tecchio 80 80125 Naples Italy martina.salzanodeluna@unina.it; b Hephaestus Laboratory, School of Chemistry, Faculty of Sciences, Democritus University of Thrace GR-65404 Kavala Greece kyzas@chem.duth.gr

## Abstract

The development of sustainable, shape-stable, and tunable adsorbents is a key challenge for water remediation, especially when pollutants differ in charge, structure, and adsorption mechanism. In this work, flame-generated soot particles were upcycled as carbonaceous fillers in crosslinked chitosan-based aerogel beads for the removal of two chemically distinct model contaminants: hexavalent chromium, Cr(vi), and Rhodamine 6G (R6G). Pristine and nitric-acid-oxidized soot were incorporated into the chitosan matrix at different ratios to investigate how the filler surface chemistry and the aerogel composition affect the adsorption performance. The resulting nanocomposite aerogels retained a porous bead morphology and displayed pH-dependent adsorption behavior. Cr(vi) removal was favored by chitosan-rich formulations under acidic conditions (highest experimental adsorption capacity around 75 mg g^−1^ obtained with the CS : soot-ox 2 : 1 sample at 40 °C), consistently with the role of protonated chitosan sites in attracting anionic chromate species. In contrast, R6G uptake increased with the content of oxidized soot (highest experimental adsorption capacity around 100 mg g^−1^ obtained with the CS : soot-ox 1 : 2 sample at 50 °C), highlighting the contribution of carbonaceous aromatic domains and oxygen-containing surface functionalities to dye adsorption. Kinetic and equilibrium adsorption measurements indicated heterogeneous adsorption behavior, well described by the Elovich and Toth models, respectively. Regeneration tests further indicated that beads containing oxidized soot preserved their removal ability over repeated cycles more effectively than neat chitosan, particularly for R6G. Overall, this study shows that soot oxidation and CS : soot ratio can be used as simple design parameters to tailor chitosan-based aerogel beads toward different classes of water pollutants, combining waste-carbon valorization with reusable biopolymer adsorbents.

## Abstract 2

Chitosan/Oxidized soot aerogels act as low-cost, regenerable adsorbents for both cationic dye (Rhodamine 6G) and toxic Cr(vi) removal.

## Graphical abstract

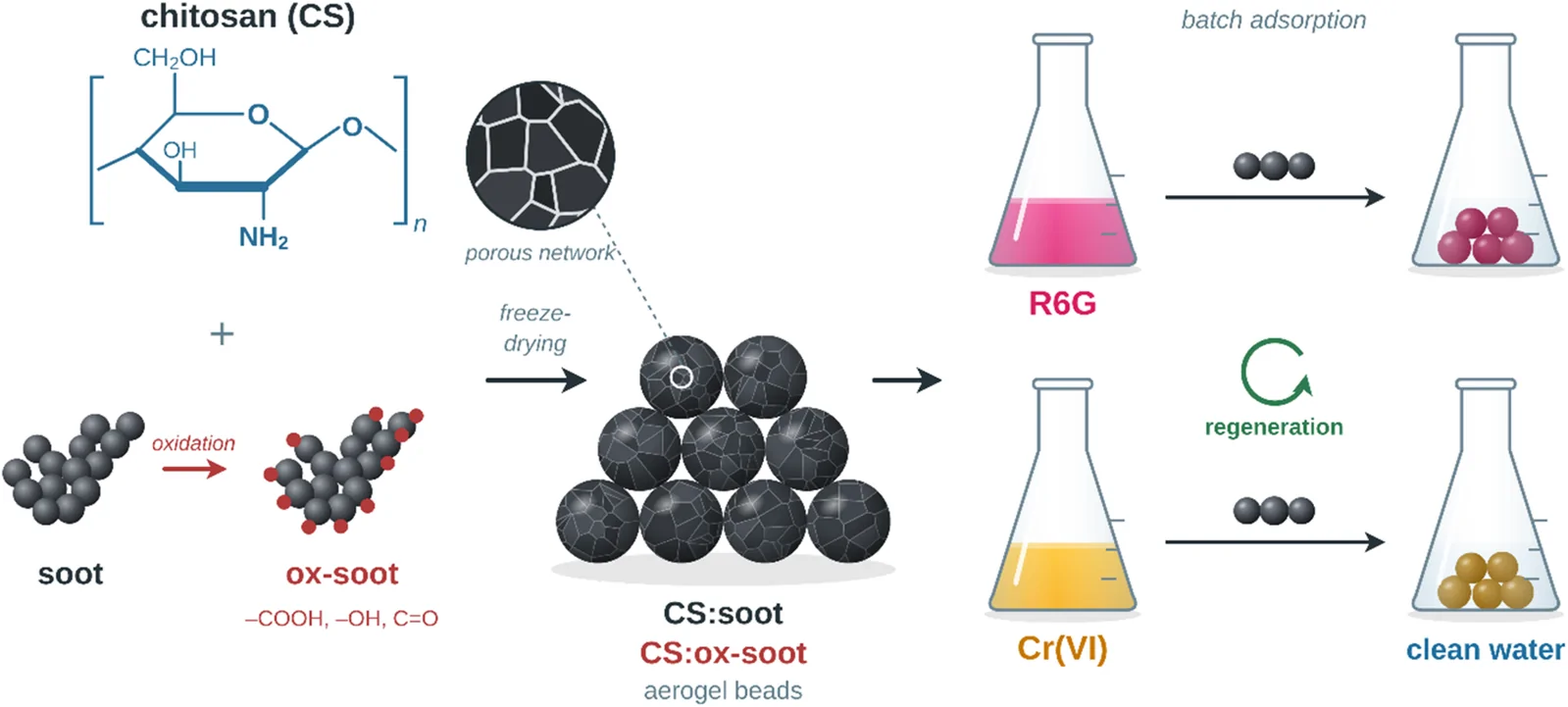

## Introduction

Industrial effluents often contain chemically diverse pollutants, among which synthetic dyes and toxic metal species are especially challenging because they differ markedly in charge, molecular structure, persistence, and removal mechanism. Synthetic organic dyes are widely used in textile, printing, paper, cosmetic, food, and biomedical sectors, and their release into water bodies can reduce light penetration, affect photosynthetic activity, alter dissolved oxygen levels, and introduce toxic or bioaccumulative compounds into aquatic ecosystems.^1^ Heavy metal contamination is equally critical because metal species are non-biodegradable and can persist, migrate, and accumulate in environmental compartments and living organisms.^2,3^

Among the available remediation strategies, adsorption stands out as a highly promising technique due to its simplicity, cost-effectiveness, high removal efficiency, and adaptability to different contaminants.^4,5^ The performance of an adsorption process largely depends on the nature and design of the adsorbent material. Indeed, the practical value of an adsorbent material depends not only on its uptake capacity, but also on its morphology, mechanical stability, ease of recovery, and regeneration. In this respect, aerogel beads are attractive because they combine the high porosity and accessibility of aerogels with easier handling and separation than powders.^6,7^ Chitosan (CS) is particularly suited for such materials because it is an abundant, biodegradable, and chemically versatile biopolymer bearing amino and hydroxyl groups that can participate in electrostatic interactions, hydrogen bonding, ion exchange, and coordination with pollutants.^8–10^ Nevertheless, pristine chitosan generally suffers from limited mechanical stability, pH-dependent swelling or dissolution, low surface area, and poor affinity toward cationic organic molecules.^11,12^ These limitations can be addressed by designing crosslinked chitosan-based nanocomposite aerogels in which the biopolymer network provides processability and pH-responsive functional sites, while a dispersed filler introduces complementary surface chemistry and adsorption pathways.^13,14^ In this sense, carbonaceous fillers are especially appealing because they introduce aromatic domains, hydrophobic regions, tunable surface chemistry, and additional interaction mechanisms with organic pollutants.^15–17^ Although traditionally regarded as a combustion-derived pollutant, soot can also be considered a low-cost nanostructured carbon material suitable for upcycling into environmental remediation systems.^18,19^ Actually, soot-based materials have already been explored for oil/water separation and organic contaminant adsorption, mainly by exploiting their hydrophobicity and carbon-rich surface.^20–22^ More recently, flame-generated soot particles were incorporated into chitosan aerogels to obtain adsorbents for the removal of organic pollutants from water, demonstrating the potential of soot valorization within biopolymer matrices.^23^ The adsorption behavior of chitosan/soot composites is expected to depend on both the amount and the surface chemistry of the carbonaceous phase. Flame-generated soot particles may differ in size, nanostructure, aromaticity, and surface functionality depending on combustion conditions and sampling position.^24–26^ Moreover, wet oxidation treatments, including nitric-acid functionalization, are commonly used to modify carbon surfaces by increasing oxygen-containing functionalities, thereby affecting wettability, dispersion, interaction with polymer matrices, and adsorption behavior.^27,28^ In chitosan/soot beads, this opens the possibility of tuning the balance between protonatable chitosan sites and soot-derived carbonaceous domains. Such a balance is expected to be particularly important for the development of high-performance adsorbents, with broad-spectrum removal ability.

This study fits in with this framework, as it provides a thorough investigation of the adsorption behavior of aerogel beads made of chitosan and pristine soot or nitric-acid-oxidized soot particles. Besides maximizing the adsorption capacity, the goal is to elucidate how the CS : soot ratio and soot surface chemistry influence the affinity of the aerogel beads toward different species. Actually, the identification of formulation–performance relationships represents the starting point for a rational design and development of advanced adsorbent materials that can effectively remove different pollutants. To this aim, hexavalent chromium (Cr(vi)) and Rhodamine 6G (R6G) have been selected as targeted contaminants.^29^ Cr(vi) is a toxic, highly soluble, and mobile heavy metal species with strong oxidizing properties, known to be mutagenic and carcinogenic even at trace levels.^30,31^ R6G is a cationic xanthene dye widely used in textiles, paper, and biological staining, valued for its stability but hazardous due to its potential toxicity, carcinogenicity, and persistence in water.^32^ Overall, the removal of Cr(vi) and R6G represents two different adsorption challenges: an inorganic, redox-active anionic contaminant and an organic, cationic aromatic dye.

## Experimental

### Materials

Chitosan powder (CS, medium molecular weight, degree of deacetylation 75–85%), poly(ethylene glycol) diglycidyl ether (PEGDGE), and Rhodamine 6G (R6G) were purchased from Sigma-Aldrich (Milan, Italy). Potassium dichromate (K_2_Cr_2_O_7_, Fluka, puriss. p.a. ≥99.0%) was used to prepare Cr(vi) stock solutions. Acetic acid, nitric acid, hydrochloric acid, sodium hydroxide and all other reagents (Sigma-Aldrich, Milan, Italy) were of analytical grade and used as received.

### Soot synthesis and functionalization

Carbonaceous soot nanoparticles were produced in a laminar atmospheric-pressure premixed ethylene flame operated under fuel-rich conditions. The cold gas velocity was set to 10 cm s^−1^ under standard temperature and pressure conditions, and the equivalence ratio was *Φ* = 2.46. The flame was stabilized on a water-cooled sintered-bronze McKenna burner (Holthuis & Associates). A stainless-steel plate was positioned 2.5 cm above the burner surface and used as a thermophoretic collection surface for the particles formed in the flame. Under these fuel-rich conditions, the flame promotes the formation of polycyclic aromatic hydrocarbons and carbonaceous nanoparticles, eventually leading to soot growth. The sampling height was selected on the basis of previous *in situ* and *ex situ* characterizations of flame-generated carbonaceous particles produced under comparable conditions.^25,26^ After deposition, the soot layer was gently scraped from the plate surface and collected as a low-density black powder. Oxidized soot was obtained by wet oxidative functionalization with nitric acid. In a typical procedure, soot powder (250 mg) was dispersed in 15 mL of concentrated nitric acid solution (67 wt%) in a round-bottom flask equipped with a reflux condenser. The suspension was magnetically stirred and kept under reflux at 85 °C for 24 h. After cooling to room temperature, the suspension was filtered under reduced pressure using hydrophilic PTFE membrane filters with 0.45 µm pore size (Omnipore). The retained solid was thoroughly washed with Milli-Q water until the filtrate reached neutral pH, in order to remove residual acid. The recovered oxidized soot was then dried at 100 °C overnight and stored in dry conditions before use. This oxidative treatment is expected to increase the amount of oxygen-containing surface functionalities on the carbonaceous particles, such as hydroxyl, carbonyl, and carboxyl groups, thereby modifying soot wettability, dispersion behavior, and compatibility with the chitosan matrix.^28^

### Preparation of nanocomposite aerogel beads

Nanocomposite aerogel beads were synthesized at different CS : soot and CS : soot-ox mass ratios, namely 2 : 1, 1 : 1, and 1 : 2. For the preparation, soot nanoparticles, either pristine or oxidized, were first pre-dispersed in a 2% v/v acetic acid aqueous solution in which a small amount of CS had been previously dissolved at a concentration of 1 mg mL^−1^ to aid the dispersion of the carbonaceous nanoparticles.^23^ After sonication for 4 h, stable high-loading aqueous dispersions were obtained. The soot concentration was set to 2.5, 5.0, or 10.0 mg mL^−1^ for the 2 : 1, 1 : 1, and 1 : 2 samples, respectively. Then, additional CS powder was added to the dispersions to reach a final total CS concentration of 5 mg mL^−1^, and the mixtures were kept under stirring for 48 h. To promote chemical crosslinking of the chitosan network, poly(ethylene glycol) diglycidyl ether (PEGDGE) was added to each mixture at 25 wt% with respect to CS and stirred for 20 min. Afterwards, the mixtures were loaded into plastic syringes and dripped into a liquid nitrogen bath with the aid of a syringe pump. The frozen beads were then collected and freeze-dried to obtain nanocomposite aerogel beads.

### Characterization of aerogel beads

The microstructure of the aerogel beads was investigated by scanning electron microscopy (SEM) using a Tescan CLARA microscope. Prior to observation, the samples were sputter-coated with a thin Au layer. Fourier transform infrared (FTIR) spectroscopy was carried out in attenuated total reflectance (ATR) mode using a PerkinElmer Spectrum 3 spectrometer. ATR spectra were recorded at a resolution of 4 cm^−1^ by averaging 64 scans. FTIR analysis was used to assess the main functional groups of chitosan, pristine soot, oxidized soot, and representative nanocomposite aerogel beads.

The adsorption behavior of the nanocomposite aerogel beads was investigated by batch adsorption tests using Cr(vi) and R6G as model pollutants. Stock solutions of Cr(vi) and R6G were prepared at 1 g L^−1^ and subsequently diluted to the desired initial concentrations. Cr(vi) solutions were prepared from K_2_Cr_2_O_7_. For all adsorption experiments, the residual pollutant concentration was determined by UV-vis spectroscopy. After each adsorption test, an aliquot of the liquid phase was withdrawn from the supernatant, filtered through a 0.45 µm nylon membrane, and analyzed. The residual concentration of Cr(vi) was determined by the 1,5-diphenylcarbazide colorimetric method, measuring the absorbance of the Cr(vi)–diphenylcarbazide complex at around 540 nm.^33^ The residual concentration of R6G was determined from the absorbance at 525 nm.^34^ Adsorption studies were performed under different experimental conditions. First, the effect of solution pH on adsorption performance was investigated. To this aim, the pH of the initial pollutant solution was adjusted by small additions of HCl or NaOH solutions. To support the interpretation of the pH-dependent adsorption behavior, the apparent point of zero charge (pH_pzc_) of the nanocomposite aerogel beads was estimated by the pH drift method.^35^ Adsorption kinetic tests were then carried out under the selected pH conditions by monitoring the residual pollutant concentration as a function of contact time. Adsorption isotherms were obtained by varying the initial pollutant concentration at fixed adsorbent dosage and pH. Isotherm tests were also performed at different temperatures to evaluate the effect of temperature on the adsorption process. Finally, the effect of adsorbent dosage was investigated by varying the amount of aerogel beads added to the pollutant solution. To complete the evaluation of the adsorption behavior of the CS : soot and CS : soot-ox aerogel beads, regeneration tests were performed on selected formulations. After each adsorption step, the beads were recovered and first washed with distilled water. They were then placed in baths at different pH values for 2 h to promote desorption, and finally washed again with water before being reused in the following adsorption cycle. Nitrogen adsorption–desorption isotherms were measured at 77 K to determine the surface area and pore size distribution of soot and soot-ox powders. Measurements were performed using a volumetric analyser (3Flex, Micromeritics). Before the measurement, the powders were pre-treated at 150 °C under a nitrogen flow for 8 hours. The specific surface area (SSA) was estimated by applying the Brunauer–Emmett–Teller (BET) model to the experimental data. The cumulative pore size and differential pore size distribution were evaluated by using the density functional theory slit kernel. Raman spectra were acquired using a Horiba XploRA Raman microscope equipped with a 100× objective (Olympus, NA 0.9), a frequency-doubled Nd:YAG laser (*λ* = 532 nm), and a 200 µm confocal pinhole. The spectrometer was calibrated using the silicon Raman band at 520 cm^−1^. Spectra collected at different locations were baseline-corrected and normalized to the G-band maximum before averaging. For graphical presentation only, the average spectra were subjected to mild Savitzky–Golay smoothing (9 points, third-order polynomial).

## Results and discussion

### Microstructural and physical characterization

Representative SEM micrographs of the aerogel beads are reported in Fig. 1.

**Fig. 1:**
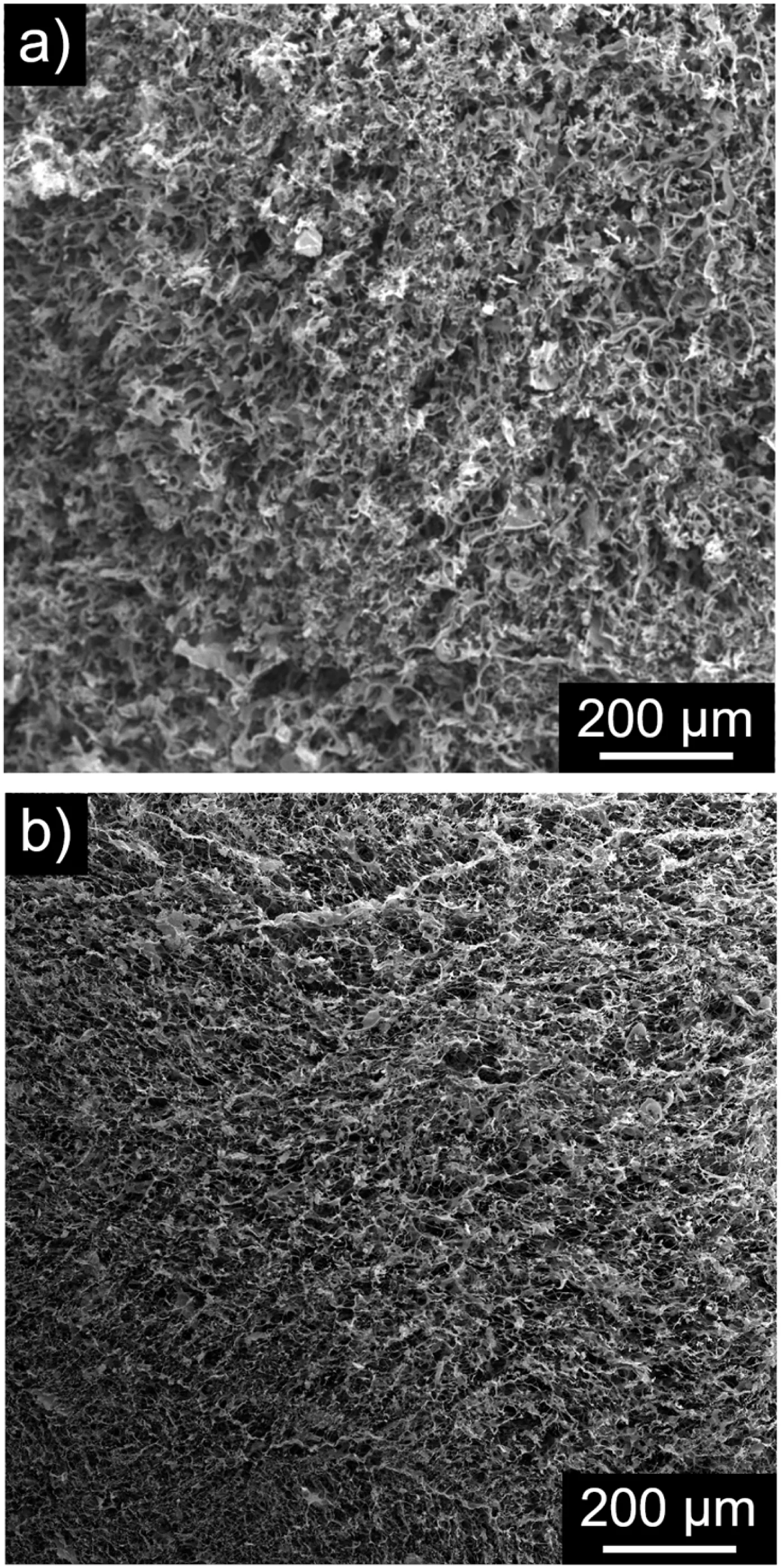
Representative SEM images showing the microstructure of (a) CS : soot 1 : 1 and (b) CS : soot-ox 1 : 1 aerogels.

The SEM images reveal significant differences in the morphology of the two aerogels. The CS : soot aerogel exhibits a highly porous three-dimensional network composed of relatively large and interconnected voids and thin pore walls, indicating a relatively loose structural organization (Fig. 1a). On the other hand, the CS : soot-ox aerogel is characterized by a more compact microstructure, with reduced pore size and a denser arrangement of the polymeric network (Fig. 1b). The pore walls appear thicker and more continuous, while the overall porosity is less pronounced than in the pristine soot sample. This morphological evolution suggests that the oxidation of soot enhances its interaction with chitosan through the introduction of oxygen-containing functional groups, promoting stronger interfacial adhesion and a more uniform distribution of the filler within the polymer matrix.

More insights into the CS-nanoparticles interactions can be gained by FTIR analysis (Fig. 2).

**Fig. 2:**
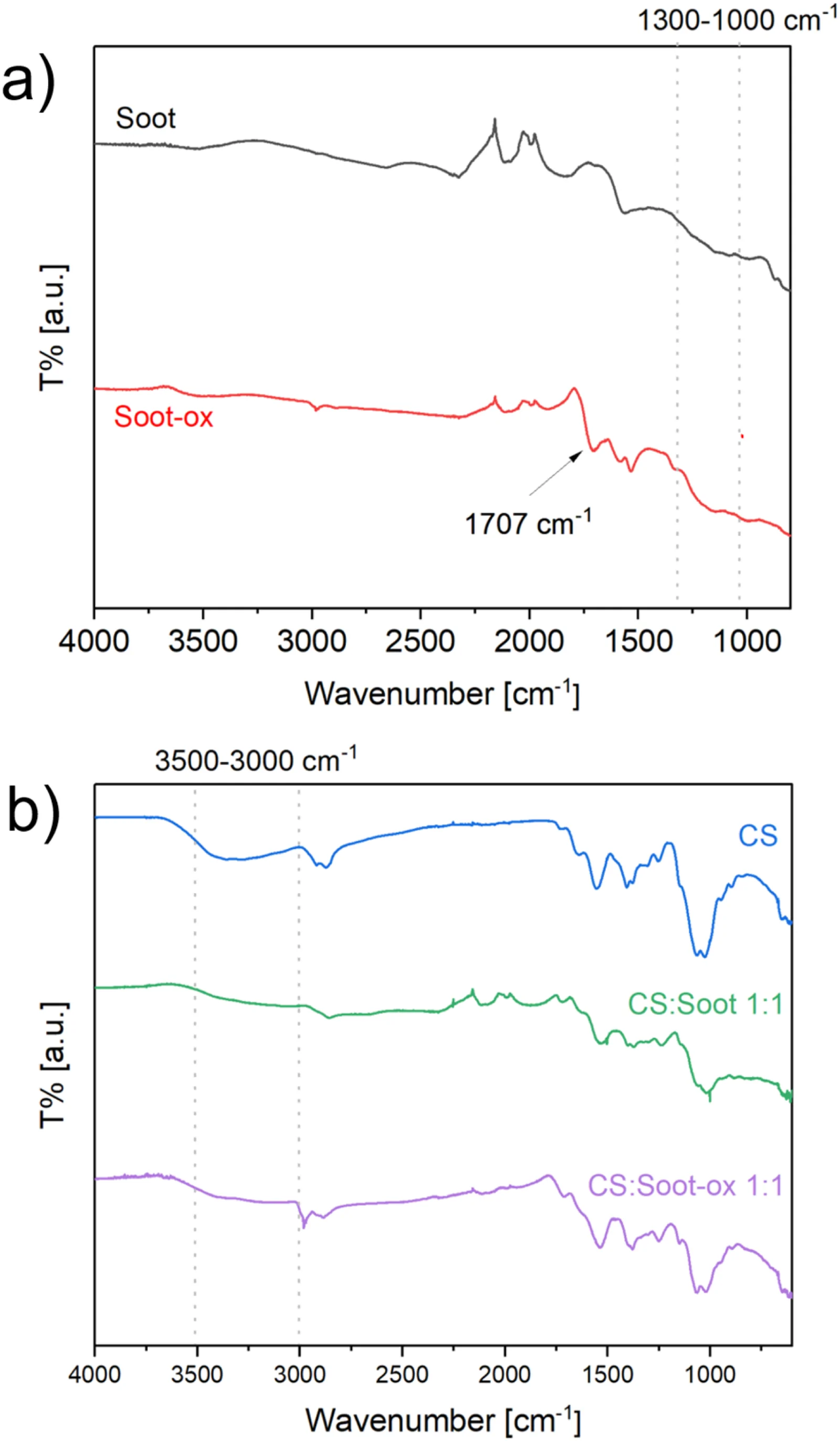
FTIR spectra of (a) soot and soot-ox (b) CS, CS : soot 1 : 1 and CS : soot-ox 1 : 1.

The FTIR spectra indicated that the soot surface was successfully modified by the oxidation treatment, which was confirmed by the appearance of oxygen-containing functional groups (Fig. 2a). In particular, the strong absorption band appearing at 1707 cm^−1^ in the spectrum of the oxidized soot indicates C=O stretching vibration of carbonyl and carboxylic acid groups. In addition, the increase in intensity in the region 1300–1000 cm^−1^ can be also ascribed to C–O stretching vibrations of phenolic, alcoholic, ether and carboxyl groups.^36^ The formation of the crosslinked composite beads was also verified by FTIR analyses (Fig. 2b). After crosslinking, the broad absorption band in the range 3500–3000 cm^−1^, which corresponds to the stretching vibrations of the O–H and N–H groups of pristine CS, is less evident in the beads, suggesting that those functional groups have been involved in the crosslinking process.^37^

FTIR analyses were carried out also on dry beads before and after the adsorption of the selected model pollutants in order to gain insights into the adsorbent–pollutant interaction (Fig. 3).

**Fig. 3:**
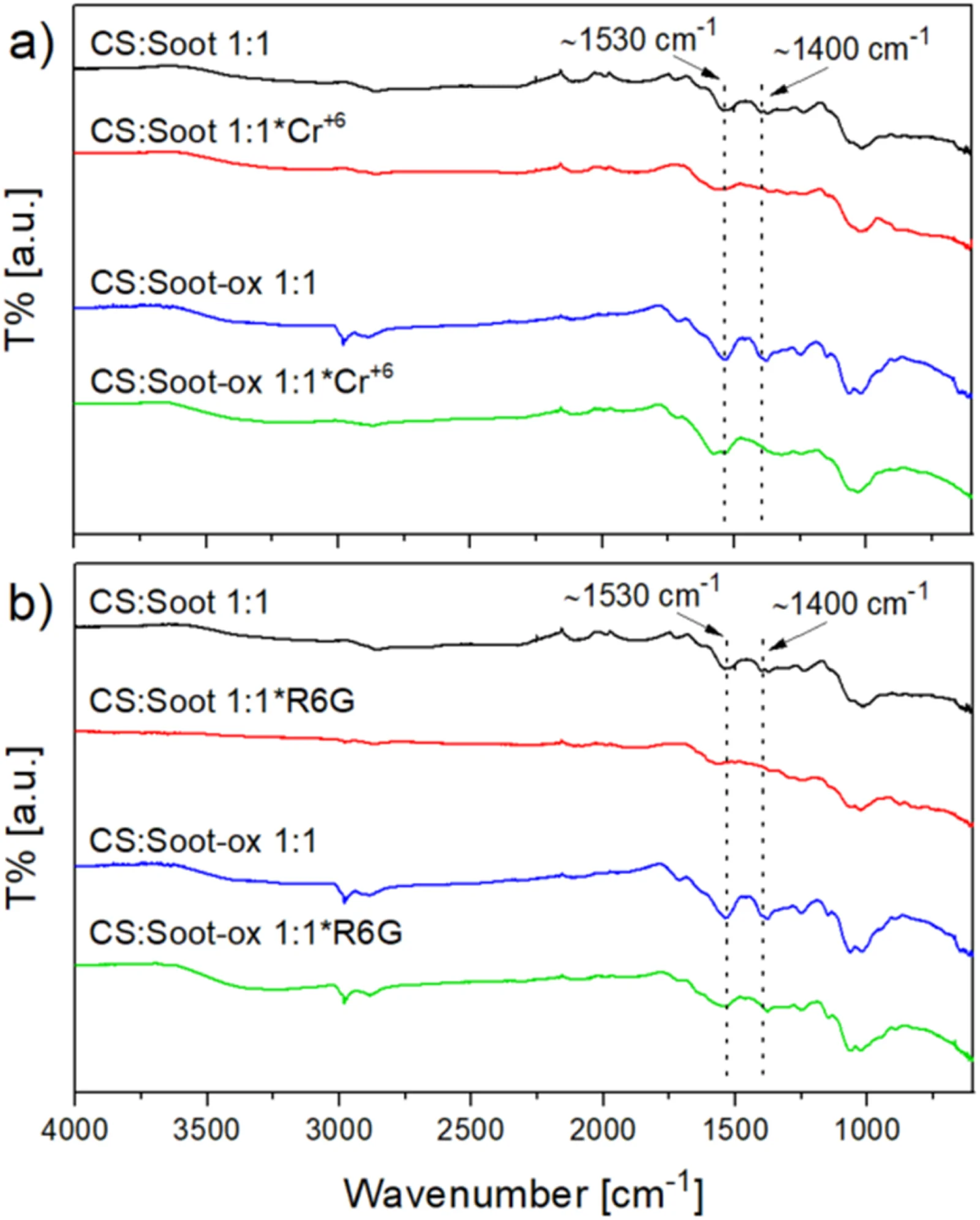
FTIR spectra of CS : soot 1 : 1 and CS : soot-ox 1 : 1 before and after adsorption of (a) Cr(vi) and (b) Rhodamine 6G.

The FTIR spectra of CS : soot 1 : 1 and CS : soot-ox 1 : 1 nanocomposite beads (taken as representative compositions) exhibited a few relevant changes in some characteristic absorption bands after adsorption of Cr(vi) and Rhodamine 6G (R6G), which indicated the participation of surface functional groups in the adsorption process. For both the CS : soot and CS : soot-ox samples, Cr(vi) adsorption resulted in a slight shift of the band near 1530 cm^−1^ toward higher wavenumbers (blue shift) and a decrease in the intensity of the band around 1400 cm^−1^, which can be related to N–H vibrations of protonated amino and residual amide groups and to CH_2_/CH_3_ deformation vibrations of chitosan, respectively (Fig. 3a). The blue shift suggests a perturbation of the –NH_3_^+^ groups upon electrostatic interaction with HCrO_4_^−^/Cr_2_O_7_^2−^ anions, and these changes appear consistent with the involvement of chitosan in Cr(vi) adsorption, possibly through electrostatic attraction and surface complexation as reported before for chitosan-based adsorbents.^38–40^ After R6G adsorption (Fig. 3b), the same bands were markedly attenuated, especially for CS : soot, while for CS : soot-ox the C–O stretching bands in the 1100–1000 cm^−1^ region shifted slightly toward lower wavenumbers (red shift), indicating that the oxygen-containing groups introduced by oxidation also take part in dye binding, likely through hydrogen bonding that weakens the C–O bonds. Since R6G is a cationic dye, its uptake can be further attributed to electrostatic attraction with the deprotonated carboxyl groups of soot-ox and to π–π stacking between its xanthene rings and the aromatic sp^2^ domains of soot.^41,42^ Overall, electrostatic attraction is further confirmed by the evaluation of the effect of pH on adsorption, along with pHpzc, which are discussed in the following sections.

The nitrogen adsorption–desorption isotherms of soot and soot-ox exhibit Type II-like profiles, characterised by a gradual increase in nitrogen uptake at intermediate relative pressures and a steep rise near saturation, without a distinct saturation plateau (Fig. 4a). According to the IUPAC classification, Type II behaviour reflects monolayer–multilayer adsorption on nonporous or macroporous surfaces.^43^ The hysteresis observed at high relative pressures is consistent with an additional contribution from capillary condensation in textural voids. Oxidation markedly decreases the BET specific surface area, from 120.1 ± 1.2 m^2^ g^−1^ for soot to 76.0 ± 0.5 m^2^ g^−1^ for soot-ox. A similar reduction in SSA was reported by Legocka *et al.* for carbon black following oxidation with a 30% aqueous hydrogen peroxide solution.^44^ The pore-size distributions reveal further differences between the samples (Fig. 4b). Both distributions display contributions at small pore widths, whereas soot-ox exhibits a more pronounced, broad contribution in the mesopore range, consistent with its greater nitrogen uptake and more pronounced hysteresis near saturation. These differences may reflect changes in the accessibility and arrangement of voids within particle aggregates following oxidation. Similarly, Pattammattel *et al.* observed a decrease in BET surface area accompanied by an increase in average pore width for carbon black following oxidation with concentrated nitric acid.^45^ Overall, the combined differences in surface functionality, SSA and pore-size distribution are therefore expected to substantially influence the adsorption performance of the corresponding chitosan/soot and chitosan/soot-ox nanocomposite aerogels.

**Fig. 4:**
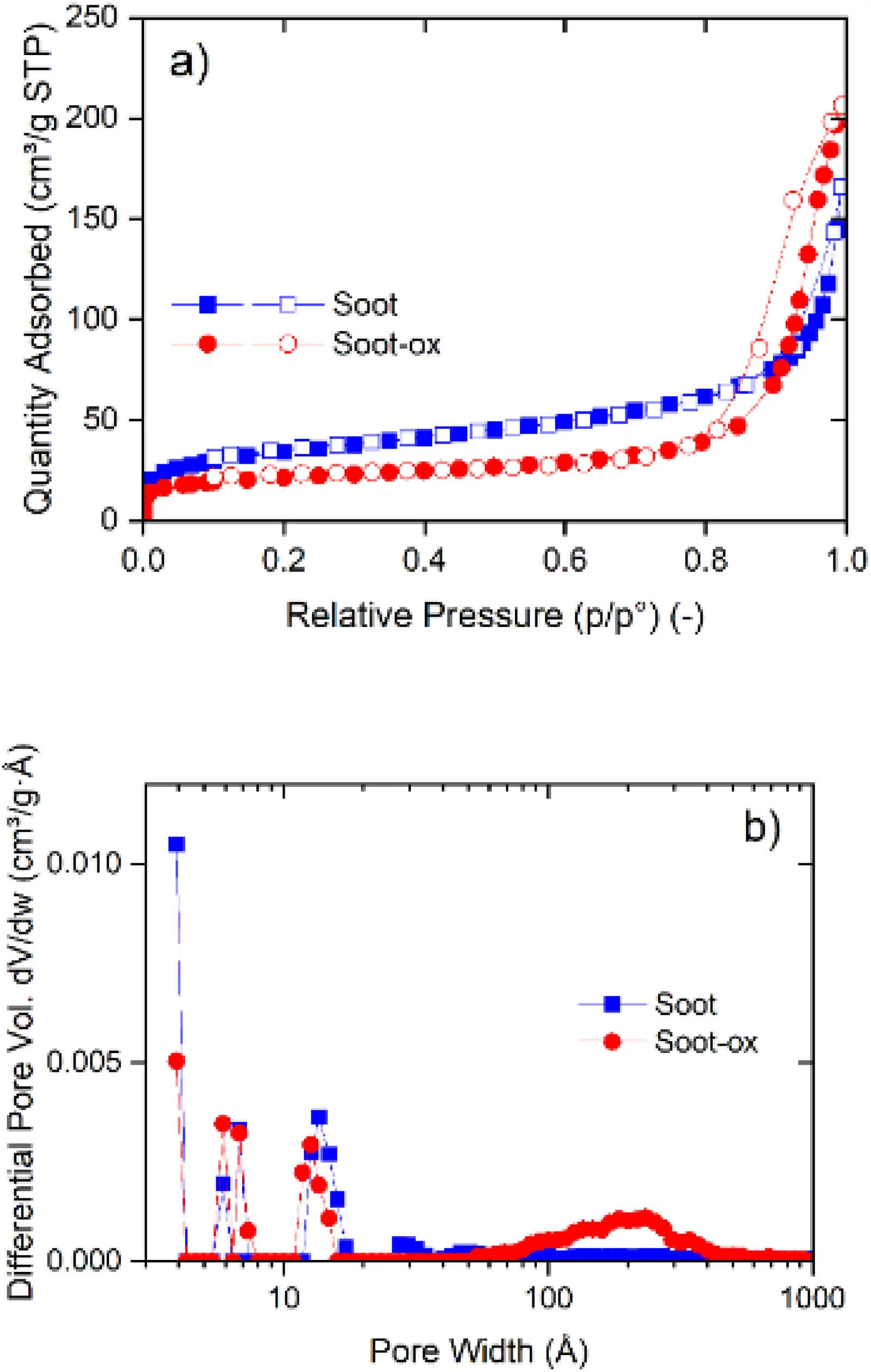
(a) N_2_ adsorption (full symbols) and desorption (empty symbols) isotherms; (b) differential pore volume distributions.

Raman spectra of soot and soot-ox exhibit the characteristic D and G bands of disordered sp^2^ carbon, at approximately 1330 and 1600 cm^−1^, respectively (Fig. 5). The *I*(D)/*I*(G) ratio increases from 0.780 ± 0.012 for soot to 0.894 ± 0.010 for soot-ox. This moderate increase is consistent with a greater contribution from defect-related Raman scattering, suggesting local modifications of the sp^2^ carbon network following oxidation. Nevertheless, the overall similarity of the spectral profiles indicates that the characteristic carbon structure is largely retained.

**Fig. 5:**
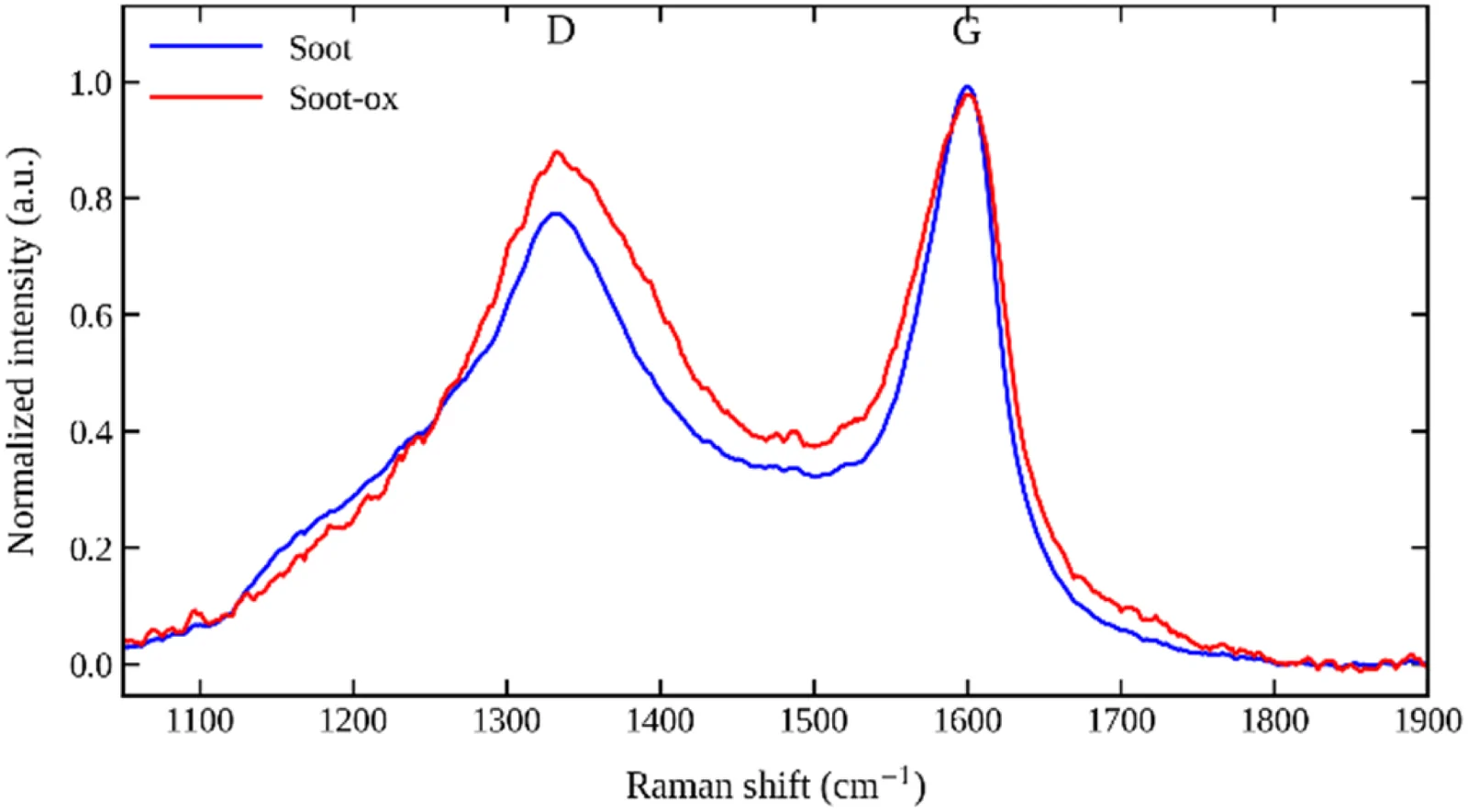
Raman spectra of pristine soot (Soot) and oxidized soot (Soot-ox). The curves represent the average spectra obtained from five and three measurements, respectively.

### Adsorption properties

The pH of the liquid medium is a critical parameter for the evaluation of adsorbent performance, since it affects both the surface chemistry of the adsorbent and the speciation/charge state of the adsorbate. After assessing the stability under different pH conditions (see Section S1 in the SI), pH-dependent adsorption capacity of CS : soot and CS : soot-ox aerogel beads was evaluated. Each experiment was performed 3 times to ensure its reliability. Fig. 6 shows the percentage removal, *R*%, of Cr(vi) and R6G, calculated as:
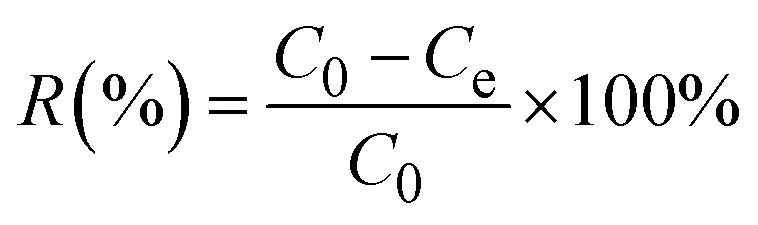
where, *C*_0_ corresponds to the initial concentration of the pollutants (mg L^−1^) and *C*_e_ is the equilibrium concentration at the end of the adsorption process (mg L^−1^).

**Fig. 6:**
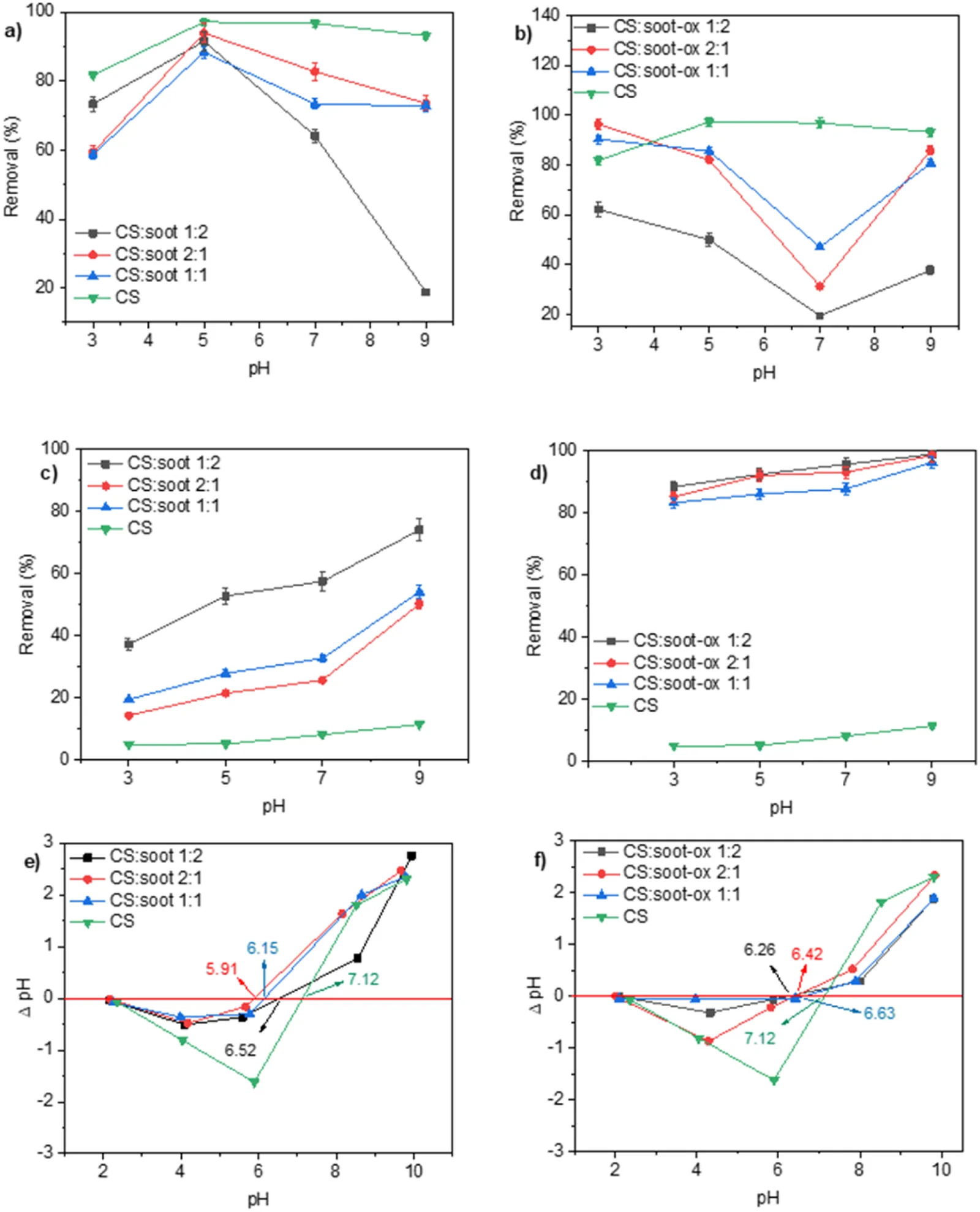
Effect of pH on the removal of (a and b) Cr(vi) (adsorbent dosage = 0.5 g L^−1^, *T* = 30 °C, *C*_0_ = 5 mg L^−1^) and (c and d) R6G (adsorbent dosage = 0.5 g L^−1^, *T* = 30 °C, *C*_0_ = 10 mg L^−1^) by using (a–c) CS : soot and (b and d) CS : soot-ox aerogel beads. Calculation of pH_pzc_ of the adsorbents by using pH change according to the drift method: (e) CS : soot and (f) CS : soot-ox aerogel beads.

In the presence of Cr(vi) (Fig. 6a and b), pristine CS aerogel exhibited the highest removal efficiency, with *R*% up to approximately 97% at pH = 5. The addition of carbonaceous nanoparticles, either pristine or oxidized, generally reduced the adsorption performance over most of the investigated pH range. Nevertheless, the highest removal efficiencies were always found under acidic conditions, where protonation of chitosan amino groups and other surface functionalities favors the electrostatic attraction of anionic Cr(vi) species. In aqueous solution, Cr(vi) mainly exists as anionic chromate/dichromate species, whose relative distribution strongly depends on pH.^46^ Therefore, the enhanced removal observed at acidic pH is consistent with the stronger attraction between positively charged/protonated sites on the aerogel surface and negatively charged chromate species. For all the investigated materials, indeed, the pH value for which neutral charge was observed (*i.e.*, pH_PZC_) is in the range ∼6–7, and thus the aerogels are positively charged below this threshold (Fig. 6e and f). In optimal conditions (*i.e.*, at pH = 5), no significant difference can be observed among the CS : soot samples, whose *R*% values essentially match that of pristine CS aerogel (Fig. 6a). On the other hand, optimal conditions for the CS : soot-ox aerogels were found at even lower pH, that is, at pH = 3, likely because of the presence of oxygen-containing groups introduced by soot oxidation. In particular, at pH 3, carboxyl groups are more protonated, potentially reducing electrostatic repulsion toward anionic Cr(vi) species which can mainly interact with protonated amino groups of chitosan, while hydroxyl and epoxy groups may provide additional interactions. In this case, *R*% values largely vary depending on the material formulation (Fig. 6b). Only the sample at lower filler content (*i.e.*, the CS : soot-ox 2 : 1) can reach removal efficacy up to ∼98%, closely followed by the CS : soot-ox 1 : 1 sample. At higher content of oxidized soot, instead, the removal of Cr(vi) is much lower than for the pristine CS aerogel. This behavior suggests that, for Cr(vi), the chitosan-rich phase remains the dominant component responsible for adsorption, while excessive soot-ox loading may dilute the amount of accessible protonatable chitosan sites and/or partially disrupt the continuity of the polymer network.

In the presence of R6G (Fig. 6c and d), the adsorption behavior was markedly different. The removal efficiency of pristine CS aerogel was very low, remaining well below 20% over the whole investigated pH range. This confirms the limited affinity of neat chitosan aerogel toward this cationic aromatic dye. Conversely, the addition of either pristine or oxidized soot significantly improved R6G removal. In both CS : soot and CS : soot-ox aerogels, the removal efficiency increased with increasing carbonaceous filler content, and this effect was much more pronounced for the oxidized-soot-containing samples. The improvement can be ascribed to the additional adsorption pathways introduced by the carbonaceous phase, including interactions between the aromatic structure of R6G and sp^2^ carbon domains, as well as polar interactions and hydrogen bonding promoted by oxygen-containing groups on oxidized soot. In addition, by increasing the pH of the solution, the *R*% values increased, likely due to stronger electrostatic attraction between the positively charged R6G (cationic dye) and the negatively charged adsorbents (see Fig. 6e and f).^47^ Hence, for the removal of R6G optimal conditions were set at pH = 9 for all the investigated aerogels.

The kinetics of the adsorption process under optimal pH conditions was then investigated. The results are reported in Fig. 7, where the amount of pollutant adsorbed over time *q*_*t*_, was calculated as:
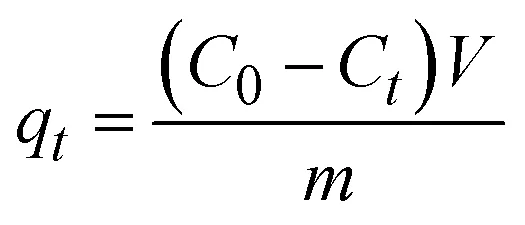
where, *C*_0_ corresponds to the initial concentration of the pollutants (mg L^−1^), *C*_*t*_ is the pollutant concentration at a given time (mg L^−1^), *V* is the volume of the dye solution (L), and *m* is the adsorbent mass (g).

**Fig. 7:**
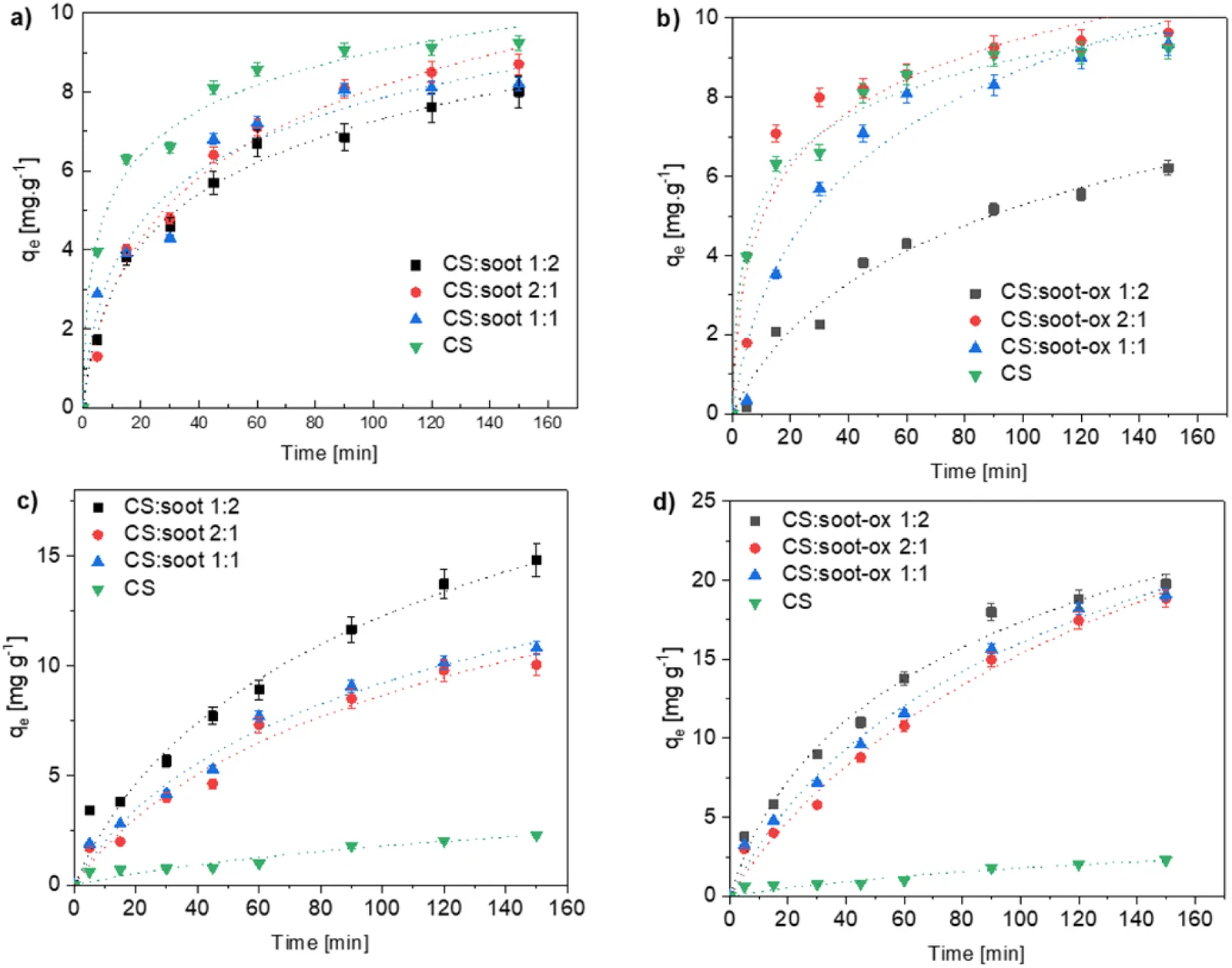
Adsorption kinetics for (a and b) Cr(vi) (adsorbent dosage = 0.5 g L^−1^, *T* = 30 °C, pH = 5, *C*_0_ = 5 mg L^−1^) and (c and d) R6G (adsorbent dosage = 0.5 g L^−1^, *T* = 30 °C, pH = 9, *C*_0_ = 10 mg L^−1^) with CS : soot and CS : soot-ox aerogels. Dashed lines correspond to best fitting to the Elovich kinetic model.

The adsorbed amount rapidly increases in the early stages for both pollutants, owing to the abundance of active sites and functional groups exposed on the surface of beads that could interact with the pollutants.^48^ At longer times, the adsorption rate steadily neared equilibrium as accessible adsorption sites on the aerogel surface were depleted and solid–liquid repulsion increased. Different kinetic adsorption models were applied to the experimental data to obtain further insight into the adsorption process. Among these, the Elovich model provided the best description of the adsorption kinetics for all the investigated systems, (see Section S2 of the SI for details on adsorption kinetic fitting). The Elovich model is an empirical equation that considers an energetically heterogeneous adsorbing surface with varying activation energy, where the adsorption rate decreases exponentially with increasing surface coverage.^49^ Accordingly, the good fit obtained here mainly reflects the heterogeneity of the bead surface, where the most accessible and energetically favorable sites are occupied first and the remaining ones become progressively less available as adsorption proceeds. However, the Elovich model does not by itself identify a specific mechanism or rate-limiting step, and the involvement of distinct steps (*e.g.*, surface binding followed by diffusion into the porous aerogel network) cannot be demonstrated on the basis of this fit alone.

Adsorption isotherms were then investigated by varying the initial pollutant concentration at fixed adsorbent dosage and pH. The equilibrium adsorption capacity *q*_e_, is reported in Fig. 8 for Cr(vi) and Fig. 9 for R6G and was calculated as:
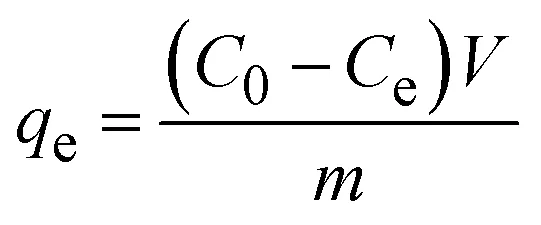
where *C*_0_ and *C*_e_ are the initial and equilibrium pollutant concentrations (mg L^−1^), respectively, *V* is the solution volume (L), and *m* is the dry mass of the adsorbent (g).

**Fig. 8:**
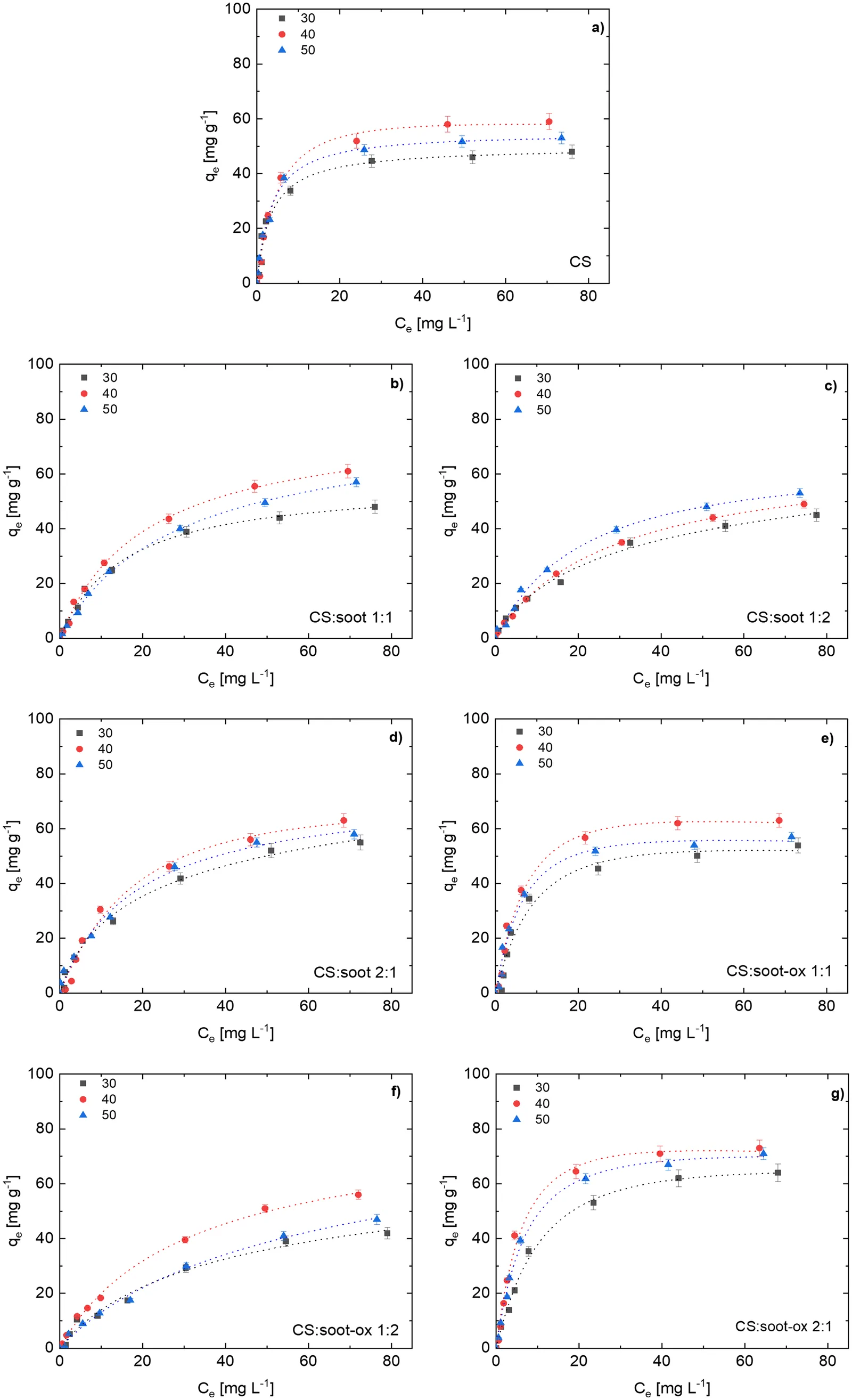
Adsorption isotherms for Cr(vi) at different temperatures with (a) CS, (b–d) CS : soot and (e–g) CS : soot-ox aerogels at different compositions (adsorbent dosage = 0.5 g L^−1^, pH = 5, contact time 150 minutes). Dashed lines correspond to best fitting to the Toth model.

**Fig. 9:**
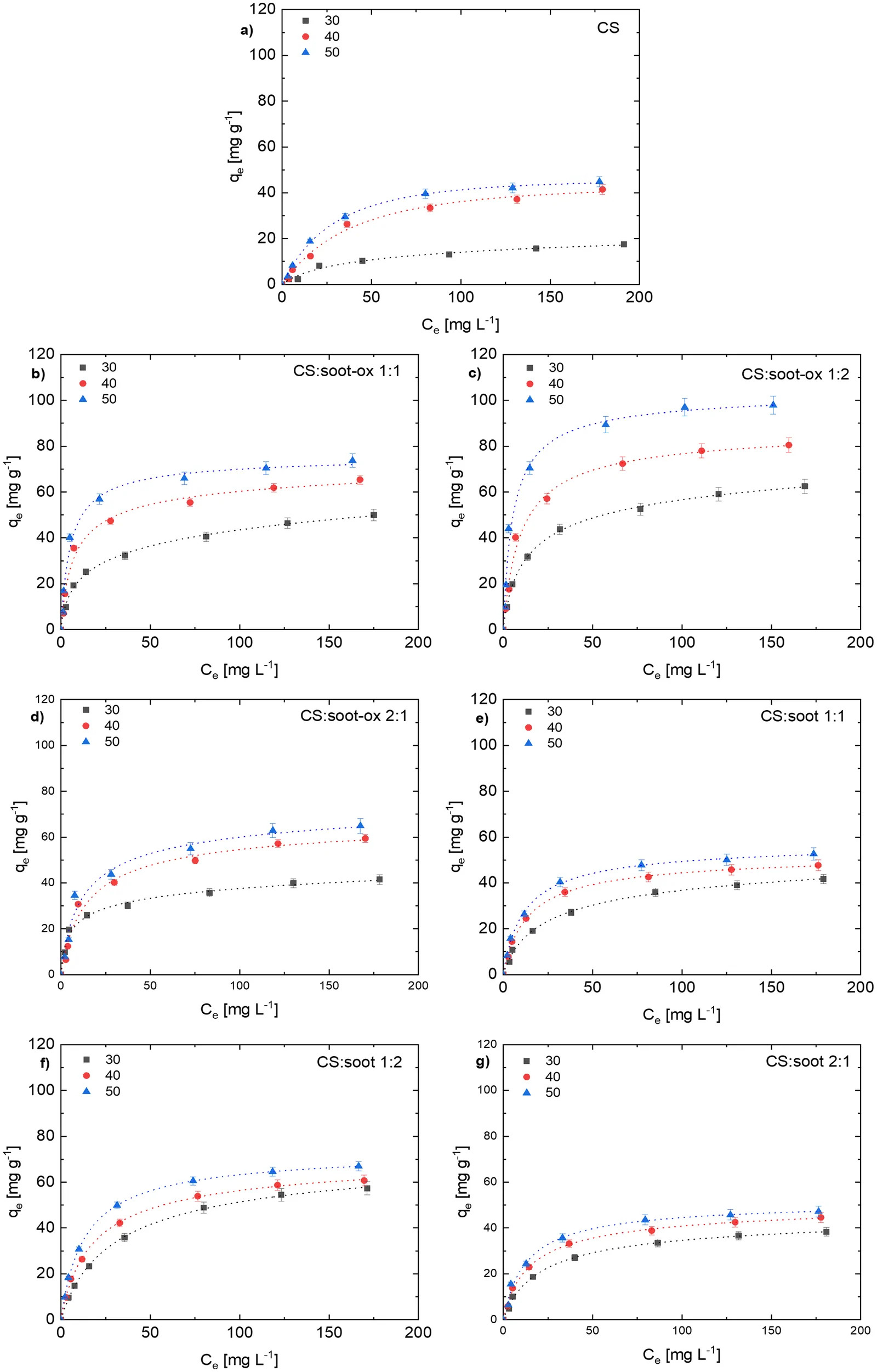
Adsorption isotherms for R6G at different temperatures with (a) CS, (b–d) CS : soot-ox and (e–g) CS : soot aerogels at different compositions (adsorbent dosage = 0.5 g L^−1^, pH = 9, contact time 150 minutes). Dashed lines correspond to best fitting to the Toth model.

For Cr(vi) adsorption (Fig. 8), increasing the temperature from 30 to 40 °C was found to enhance adsorption across all the investigated materials, likely due to an endothermic activation process. Above 40 °C, however, thermal agitation and/or weakened interactions reduce pollutant uptake. This trend may be explained by the higher solubility of the pollutant in the solvent at elevated temperatures, resulting from stronger interactions with solvent molecules and increased formation of water clusters. Additionally, at higher temperatures, the energy of the adsorbate molecules increases, making it easier for pollutant molecules to desorb from the adsorbent surface. This behavior is typical of physisorption.^50^ Indeed, at higher temperature many chitosan amino/hydroxyl-Cr(vi) chelation or CS/soot interactions are impaired, reducing active adsorption sites. Focusing on the adsorption isotherms in the optimal temperature conditions (*i.e.*, 40 °C), it turns out that the addition of relatively low amounts of carbonaceous nanoparticles to the chitosan matrix (2 : 1 samples) improves the uptake of Cr(vi), and the effect is more evident when oxidized soot is considered. Instead, the adsorption behavior of the 1 : 1 samples is comparable to that of the pristine CS aerogel, while at higher filler contents (1 : 2 samples) the Cr(vi) removal is even worse. Overall, by comparing all the investigated materials and formulations, the highest experimental adsorption capacity for Cr(vi) (around 75 mg g^−1^) was obtained with the CS : soot-ox 2 : 1 sample at 40 °C.

Different isotherm models were fitted to the experimental adsorption data to gain further insight into the process, and the Toth model^8,51,52^ was found to be the most suitable in describing the data (see Section S3 of the SI for details on adsorption isotherms fitting). The Toth model is a semi-empirical model that corrects departures from the Langmuir isotherm at both low and high concentrations, resulting in a better match to real-world systems. The model implies that adsorption favors monolayer coverage, albeit with varied site affinities due to surface heterogeneity.^53^

For R6G removal (Fig. 9), the adsorption capacity of all the investigated materials steadily increases as the temperature is raised, suggesting an endothermic process. At each temperature, and more evidently in the optimal temperature conditions (*i.e.*, 50 °C), the addition of either pristine or oxidized soot improves the adsorption of the aerogels with respect to the pure CS sample. Again, the effect is more pronounced when oxidized soot is used, and the enhancement of the R6G uptake capacity monotonically increases with the amount of filler. In this case, the highest experimental adsorption capacity for R6G (around 100 mg g^−1^) was obtained with the CS : soot-ox 1 : 2 sample at 50 °C. Again, for all the investigated materials, the experimental data are well described by the Toth model, which is consistent with adsorption on energetically non-uniform sites.

Finally, the effect of the adsorbent dosage was investigated as it influences the distribution and arrangement of adsorbate molecules on the available adsorbent surface. The percentage removal of Cr(vi) or R6G for different adsorbent dosages is shown in Fig. 10 (values in terms of equilibrium adsorption capacity are given in Fig. S2).

**Fig. 10:**
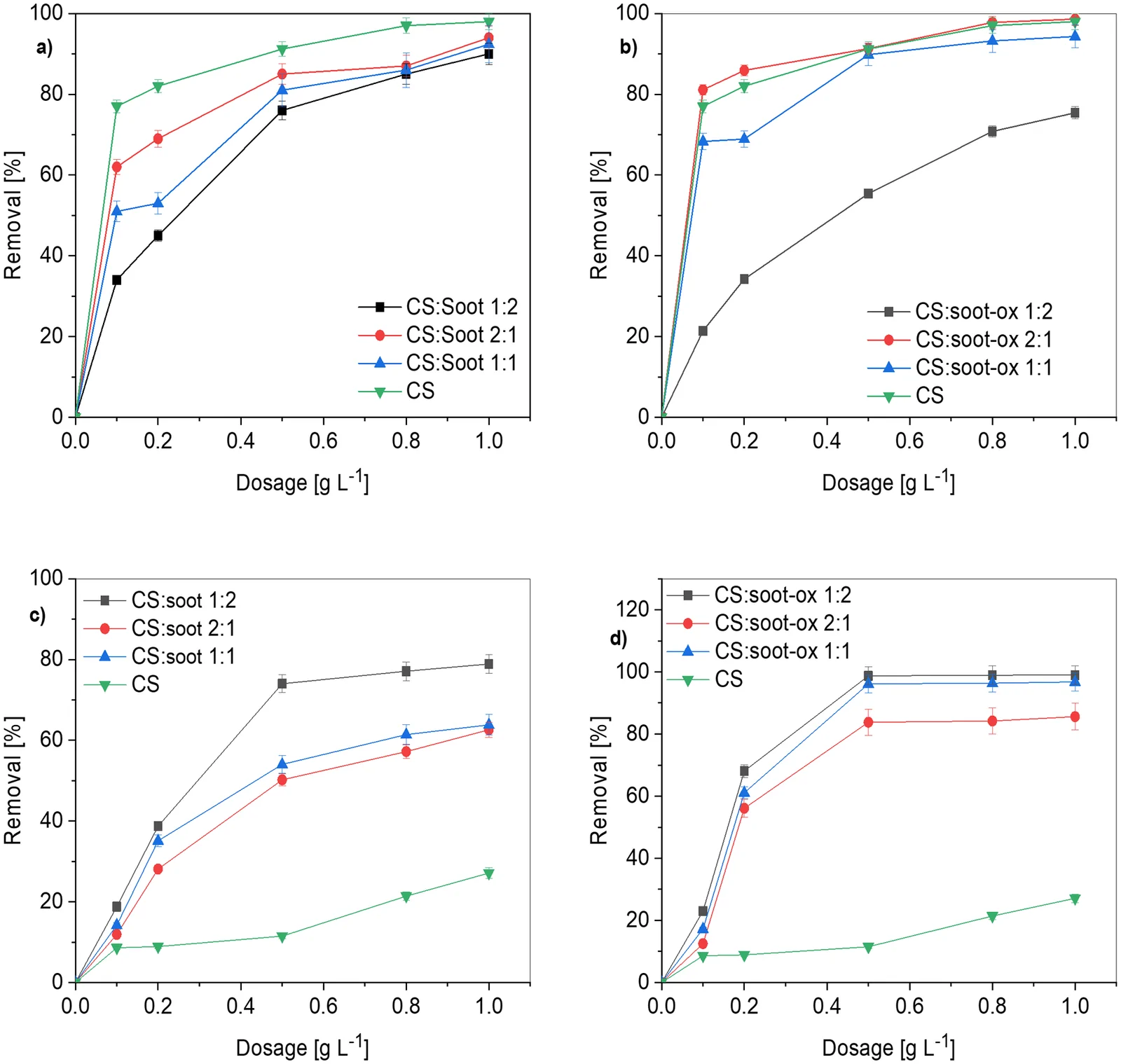
Effect of adsorbent dosage on the removal of (a and b) Cr(vi) and (c and d) R6G (*T* = 30 °C, contact time 150 minutes, pH = 5 for Cr(vi) and pH = 9 for R6G) with (a–c) CS : soot and (b–d) CS : soot-ox aerogels at different compositions. Dashed lines are a guide for the eye.

Increasing the solid-to-liquid ratio led to an increase in pollutant removal because of the larger number of accessible adsorption sites available in the system.^54^ However, above a certain threshold such an increase starts to level off, either because *R*% values reach saturation (*i.e.*, *R*% ∼100%) or because of the unfavorable adsorbent–adsorbent interactions. For Cr(vi) removal, the optimal adsorbent dosage, that is, when the maximum efficiency was achieved, is around 1 g L^−1^ for most of the investigated materials (Fig. 10a and b). Interestingly, in the case of R6G, the nanocomposites made of pristine soot needed as low as 0.8 g L^−1^ of solid-to-liquid ratio to reach the maximum performance (Fig. 10c). Even better performance is exhibited by the nanocomposites made of oxidized soot, which reached the maximum removal efficiency at around 0.5 g L^−1^ (Fig. 10d).

Overall, the thorough study of the adsorption behavior revealed that oxidized soot is preferable for enhancing the properties of chitosan-based aerogels. In particular, the CS : soot-ox 2 : 1 aerogel provides the highest improvement of the removal capacity for Cr(vi), together with a higher stability over the entire pH range, while the CS : soot-ox 1 : 2 aerogel ensures the highest uptake capacity when R6G is considered. These formulations were thus selected as the best-performing adsorbents and were thus employed for reusability tests, evaluated over multiple adsorption/desorption cycles. The results are reported in Fig. 11, in which the behavior of pristine CS aerogels is also shown as a reference.

**Fig. 11:**
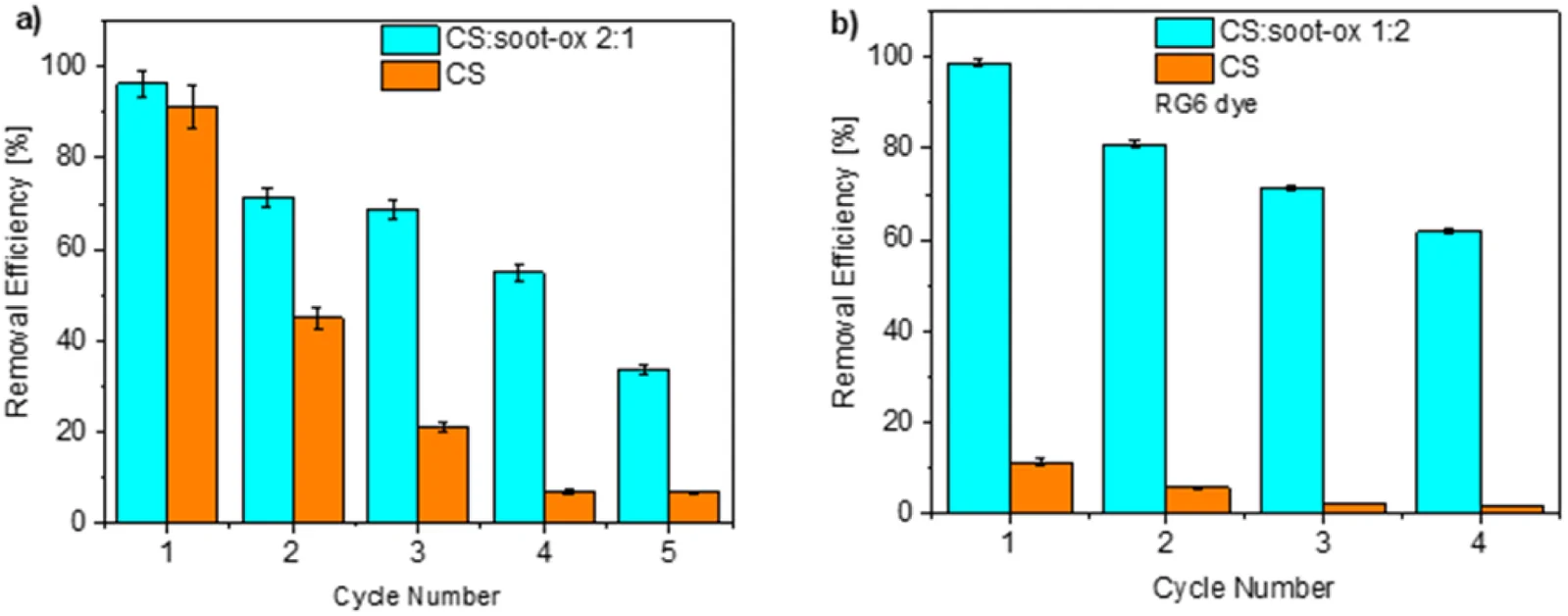
Removal of (a) Cr(vi) using CS : soot-ox 2 : 1 aerogel beads and (b) R6G using CS : soot-ox 1 : 2 aerogel beads over multiple adsorption/desorption cycles (adsorbent dosage = 0.5 g L^−1^, *T* = 30 °C, contact time = 150 minutes, pH = 5 for Cr(vi) and pH = 9 for R6G). In each plot, data for neat CS aerogel beads are reported for comparison.

For Cr(vi), the difference in removal efficiency between CS : soot-ox 2 : 1 and neat CS was relatively small during the first adsorption cycle (Fig. 11a), with removal of ∼96% and ∼91%, respectively. However, the nanocomposite beads retained their pollutant removal capacity more effectively over four consecutive cycles: after the fourth cycle, CS : soot-ox 2 : 1 still removed ∼55% of Cr(vi), corresponding to ∼57% of its initial removal efficiency, whereas neat CS dropped to ∼7% (∼8% retention), indicating improved suitability for regeneration and reuse. This behavior suggests that the incorporation of a limited amount of oxidized soot improves the robustness of the bead network and/or the stability of the active sites during adsorption/desorption.^7,23,55^ For R6G, the benefit of the nanocomposite formulation was already evident from the first adsorption cycle (Fig. 11b), with ∼99% removal for CS : soot-ox 1 : 2 compared with ∼11% for neat CS. The CS : soot-ox 1 : 2 beads preserved this advantage over four cycles, still removing ∼62% of R6G after the fourth cycle (∼63% of the initial removal efficiency), while removal by neat CS fell below 2%. This confirms that the oxidized-soot-rich formulation provides stable and reusable adsorption sites for R6G, likely associated with the combination of carbonaceous domains, oxygen-containing functionalities, and improved interfacial compatibility with the chitosan matrix.

In this framework, the pH of the eluent medium has a great effect on the desorption efficiency of Cr(vi) and R6G (Fig. 12). For Cr(vi), desorption from CS : soot-ox 2 : 1 beads increased gradually from pH 3 to pH 11 and the maximum recovery was attained under strongly alkaline conditions (Fig. 12a).^55^ This behaviour is attributed to the deprotonation of surface functional groups at high pH that weakens the electrostatic attraction and surface complexation of chromate species with the adsorbent. On the other hand, R6G showed the maximum desorption rate from CS : soot-ox 1 : 2 beads at pH 3, with a decrease as the pH increased (Fig. 12b). The acidic medium favors the protonation of the adsorbent surface, reducing its affinity to the cationic dye and facilitating the release of the dye.^56^ For both Cr(vi) and R6G, the pH-dependent desorption efficiency correlates well with the picture emerging from pH-dependent adsorption data (Fig. 6). Also, the desorption efficiencies of the CS : soot-ox nanocomposites were always higher than that of pristine chitosan, especially for the soot-ox-rich sample adsorbing R6G, proving that the oxygen-containing functional groups of soot play a relevant role in the process.

**Fig. 12:**
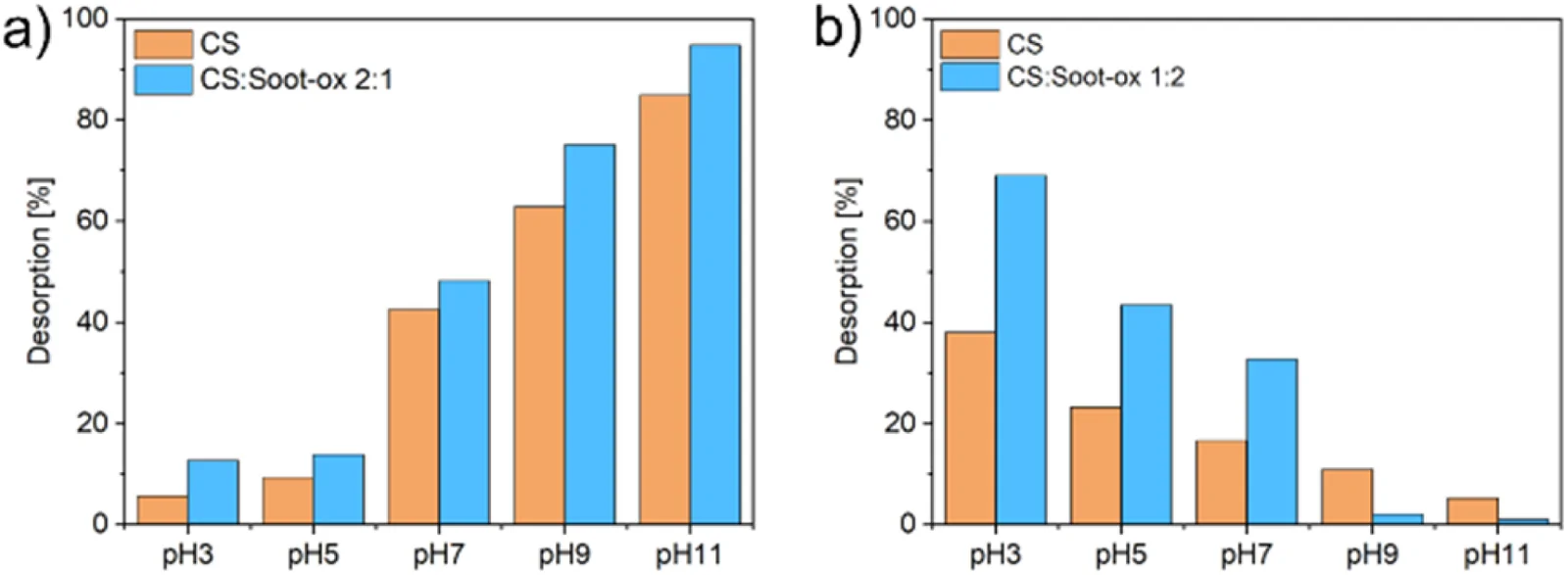
Effect of eluent pH on the desorption of (a) Cr(vi) from CS : soot-ox 2 : 1 aerogel beads and (b) R6G from CS : soot-ox 1 : 2 aerogel beads.

Finally, the maximum adsorption capacities of the optimized CS : soot-ox formulations are compared to those of other adsorbents in Table 1, which were obtained through adsorption isotherm experiments. Although certain materials have higher adsorption capacities compared to the aerogel beads proposed in the present work, their practical application could often be limited due to factors such as cost, complex synthesis procedures and difficult recovery processes. Differently, soot nanoparticles are an abundant combustion by-product that is here valorized by developing millimeter-sized aerogel beads that also allow overcoming some practical limitations in adsorbent exploitability.

**Table 1:** Comparison of the maximum adsorption capacity *Q*_max_ with literature data on comparable systems

Adsorbent materials	Type	Pollutant	*Q*_max_ [mg g^−1^]	Ref.
Chitosan-g-(*N*-vinyl pyrrolidone)/montmorillonite	Hydrogel	R6G	∼37	41
Poly(styrene)-poly(styrene-methacrylic acid) (PSt-P(St-MAA))	Microspheres	R6G	∼214	57
Silk-g-*ε*-caprolactone	Fibroin	Cr(vi)	∼325	58
		R6G	∼436	
Graphene oxide/chitosan/α-FeO(OH)	Microspheres	Cr(vi)	∼63	59
CS-GO aerogel	Microspheres	Cr(vi)	∼287	60
		MB	∼599	
Biomass carbon dots/chitosan/TiO_2_	Hydrogel	Cr(vi)	∼22	61
CS : soot-ox 1 : 2	Beads	Cr(vi)	∼58	This work
		R6G	∼100	
CS : soot-ox 2 : 1	Beads	Cr(vi)	∼75	This work
		R6G	∼67	

## Conclusions

Flame-generated soot particles were successfully incorporated into PEGDGE-crosslinked chitosan aerogel beads, obtaining lightweight and handleable adsorbents for Cr(vi) and Rhodamine 6G removal. The comparison between pristine and oxidized soot showed that the adsorption performance is not simply governed by the amount of carbonaceous filler, but by the balance between the chitosan network and the soot-derived surface chemistry. The best formulation depended strongly on the target pollutant. Cr(vi) removal was favored by chitosan-rich beads, with CS : soot-ox 2 : 1 showing the best performance among the nanocomposites (highest experimental adsorption capacity around 75 mg g^−1^ obtained at 40 °C), consistently with the dominant role of protonated chitosan sites under acidic conditions. Conversely, R6G removal increased with the soot-ox content, reaching the highest uptake for CS : soot-ox 1 : 2 (highest experimental adsorption capacity around 100 mg g^−1^ at 50 °C), where carbonaceous aromatic domains and oxygen-containing functionalities likely provide additional interactions with the cationic dye. Kinetic and equilibrium analyses indicated heterogeneous adsorption behavior, well described by the Elovich and Toth models, respectively. Regeneration tests further showed that oxidized-soot-containing beads retained their removal ability over repeated cycles better than neat chitosan, especially for R6G. These results indicate that soot oxidation and CS : soot ratio are effective parameters to tune chitosan-based aerogel beads toward different classes of pollutants. Further work should include post-adsorption chromium speciation and column tests to better assess the mechanisms and practical applicability of the materials.

## Author contributions

Shehryar Ahmad: writing – review & editing, writing – original draft, methodology, investigation. Konstantinos N. Maroulas: writing – review & editing, writing – original draft, methodology, investigation. Mariano Sirignano: writing – review & editing, investigation. Martina Salzano de Luna: writing – review & editing, writing – original draft, methodology, investigation, supervision. George Z. Kyzas: writing – review & editing, writing – original draft, methodology, investigation, supervision.

## Conflicts of interest

There are no conflicts to declare.

## Supplementary Material

RA-OLF-D6RA09330B-s001

## Data Availability

The data supporting this article have been included as part of the supplementary information (SI). Supplementary information is available. See DOI: https://doi.org/10.1039/d6ra09330b.
