# Supplementary material for: The role of soot oxidation on the optimization of chitosan/soot aerogel beads for heavy metals and dyes adsorption
Source: RSC Adv. 2026 Nov 2. Online ahead of print. doi: 10.1039/d6ra09330b (PMC13726629; doi:10.1039/d6ra09330b)
Supplement: Supplementary file 1 [file RA-OLF-D6RA09330B-s001.pdf]

Supplementary Information (SI) for RSC Advances.
This journal is © The Royal Society of Chemistry

The role of soot oxidation on the optimization of chitosan/soot aerogel beads for heavy metals and dyes adsorption

Shehryar Ahmad,[a,1] *Konstantinos N. Maroulas,*[b,1] *Mariano Sirignano,*[a] *Martina Salzano de Luna,*[a,*] *George Z. Kyzas*[b,*]

[a] Department of Chemical, Materials, and Production Engineering (INSTM Consortium–UdR Naples), University of Naples Federico II, P.le Tecchio 80, 80125 Naples, Italy

[b] Hephaestus Laboratory, School of Chemistry, Faculty of Sciences, Democritus University of Thrace, GR-65404 Kavala, Greece

[1] These authors contributed equally

*Corresponding authors: G. Z. Kyzas (kyzas@chem.duth.gr), M. Salzano de Luna (martina.salzanodeluna@unina.it)

TABLE OF CONTENTS

S1. pH-dependent aerogel stability

S2. Fitting of adsorption kinetics

S3. Fitting of adsorption isotherms

S4. Equilibrium adsorption capacity for the effect of adsorbent dosage

S1. pH-dependent aerogel stability

The behavior of the aerogels in water was evaluated by pH-dependent stability tests, which are relevant for adsorption and regeneration processes involving aqueous media. Dried samples were soaked for 24 h in aqueous solutions at different pH values, in the range from 3 to 11 (±0.1). After immersion, the samples were recovered and dried, and the residual mass was measured. The mass loss was then used as an indicator of bead stability under acidic, neutral, and alkaline conditions. The results are shown in Figure S1.

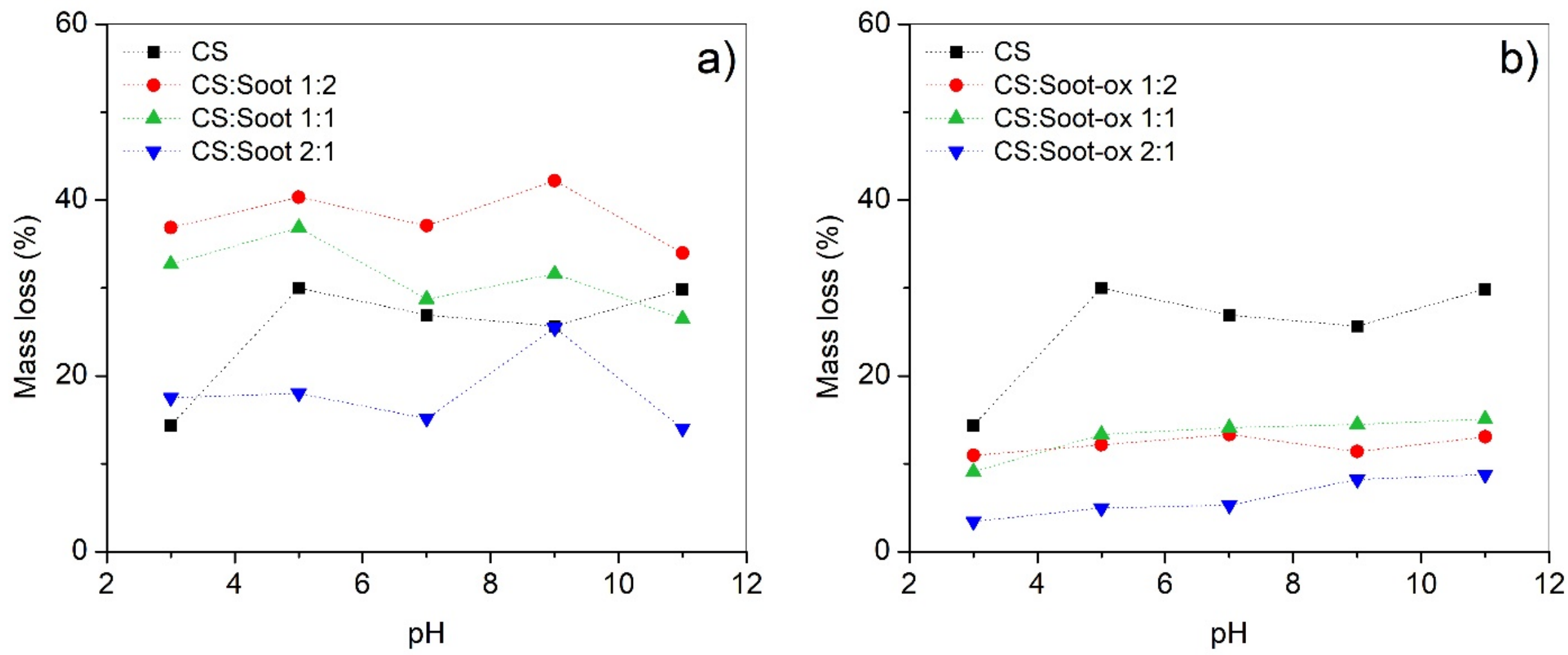

Figure S1. Stability of aerogel beads in different pH conditions: a) CS:Soot and b) CS:Soot-ox aerogels.

Neat CS beads show a marked pH-dependent mass loss, especially under alkaline conditions. This behavior can be ascribed to pH-induced changes in the chitosan network, including deprotonation, swelling/erosion, and weakening of polymer–polymer interactions. For the nanocomposite aerogels,

irrespective of the type of soot, the 2:1 composition shows the lowest mass loss, suggesting that a moderate content of carbonaceous nanoparticles can be incorporated into the chitosan network without compromising bead integrity. Conversely, at higher filler contents, namely 1:1 and 1:2, the nanoparticles likely interfere with the continuity of the polymer matrix, resulting in a higher extent of leaching or structural weakening. This trend is consistent with previous studies on chitosan-based composite aerogels, where the final stability and adsorption performance were shown to depend strongly on the balance between polymer network continuity, filler loading, and interfacial interactions. Interestingly, the CS:Soot-ox beads display slightly improved stability over a broader pH range compared with the corresponding CS:Soot samples. This effect may be related to the higher amount of oxygen-containing functionalities on oxidized soot, which can promote more favorable interactions with the chitosan matrix and contribute to the integrity of the nanocomposite network.

S2. Fitting of adsorption kinetics

The experimental adsorption kinetics data was fitted to the pseudo-first order (PFO), pseudo-second order (PSO), pseudo first order, pseudo second order, Elovich, and intra particle diffusion kinetic models (Equations S1-4, respectively) [R1]:

$$q_t = q_e(1 - e^{-k_1 t}) \quad \text{(S1)}$$

$$q_t = \frac{q_e^2 k_2 t}{1+q_e k_2 t} \quad \text{(S2)}$$

$$q_t = \frac{1}{\square}\ln(1 + \square\square t) \quad \text{(S3)}$$

$$q_t = k_{ip}\sqrt{t} + c_{ip} \qquad (S4)$$

where k_1 and k_2 are rate constant for the PFO and PSO kinetic models, respectively, α and □ represent the initial adsorption rate and the desorption constant according to the Elovich model, k_{ip} and c_{ip} correspond to the coefficients of intraparticle diffusion model.

Table S1. Best fitting parameters for the PFO kinetic model.

Cr(VI)			
**Adsorbent**	q_e **[mg g⁻¹]**	k_1 **[min⁻¹]**	**Adj**R^2
CS:Soot 1:2	7.58	3.54E-02	0.9742
CS:Soot 1:1	8.16	3.7E-02	0.9330
CS:Soot 2:1	8.63	3.10E-02	0.9873
CS:Soot-ox 1:2	6.49	1.79E-02	0.9791
CS:Soot-ox 1:1	9.28	3.06E-02	0.9861
CS:Soot-ox 2:1	9.18	7.15E-02	0.9665
**R6G**			
**Adsorbent**	q_e **[mg g⁻¹]**	k_1 **[min⁻¹]**	**Adj**R^2
CS:Soot 1:2	16.55	1.42E-02	0.9653
CS:Soot 1:1	12.07	1.52E-02	0.9815
CS:Soot 2:1	11.76	1.39E-02	0.9777
CS:Soot-ox 1:2	21.13	1.85E-02	0.9831
CS:Soot-ox 1:1	22.33	1.31E-02	0.9852
CS:Soot-ox 2:1	24.36	1.01E-02	0.9863

Table S2. Best fitting parameters for the PSO kinetic model.

Cr(VI)			
Adsorbent	q_e [mg g^{-1}]	k_2 [g mg^{-1} min^{-1}]	AdjR^2
CS:Soot 1:2	9.05	4.55-03	0.9897
CS:Soot 1:1	9.51	4.95-03	0.9474
CS:Soot 2:1	10.57	3.16-03	0.9921
CS:Soot-ox 1:2	8.90	1.67-03	0.9793
CS:Soot-ox 1:1	11.67	2.58-03	0.9777
CS:Soot-ox 2:1	10.47	8.32-03	0.9595
R6G			
Adsorbent	q_e [mg g^{-1}]	k_2 [g mg^{-1} min^{-1}]	AdjR^2
CS:Soot 1:2	23.15	4.97E-4	0.9697
CS:Soot 1:1	17.01	7.11E-4	0.9817
CS:Soot 2:1	17.07	6.13E-4	0.9757
CS:Soot-ox 1:2	28.37	5.71E-4	0.9847
CS:Soot-ox 1:1	32.18	3.13E-4	0.9865
CS:Soot-ox 2:1	37.33	1.88E-4	0.9863

Table S3. Best fitting parameters for the Elovich kinetic model.

Cr(VI)			
Adsorbent	α [mg g^{-1} min]	β [g mg^{-1}]	AdjR^2
CS:Soot 1:2	0.63	4.73E-01	0.9916
CS:Soot 1:1	0.92	4.96E-01	0.9564
CS:Soot 2:1	0.53	3.75E-01	0.9865
CS:Soot-ox 1:2	0.15	3.85E-01	0.9771
CS:Soot-ox 1:1	0.46	3.15E-01	0.9592
CS:Soot-ox 2:1	2.01	4.83E-01	0.9175
R6G			
Adsorbent	α [mg g^{-1} min]	β [g mg^{-1}]	AdjR^2
CS:Soot 1:2	0.03	5.4E-01	0.8893
CS:Soot 1:1	0.32	1.3E-01	0.9743

CS:Soot 2:1	0.24	1.8E-01	0.9817
CS:Soot-ox 1:2	0.58	1.1E-01	0.9857
CS:Soot-ox 1:1	0.37	9.1E-02	0.9878
CS:Soot-ox 2:1	0.28	7.3E-02	0.9862

Table S4. Best fitting parameters for the Intraparticle diffusion kinetic model.

Cr(VI)			
**Adsorbent**	k_{dif} **[mg g^{-1} min$^{-1/2}$]**	**c [mg g^{-1}]**	**AdjR^2**
CS:Soot 1:2	0.65	7.05E-01	0.9176
CS:Soot 1:1	0.67	1.06	0.9043
CS:Soot 2:1	0.75	4.80E-01	0.9427
CS:Soot-ox 1:2	0.55	3.18E-01	0.9621
CS:Soot-ox 1:1	0.85	1.50E-01	0.9034
CS:Soot-ox 2:1	0.76	1.86	0.7593
**R6G**			
**Adsorbent**	k_{dif} **[mg g^{-1} min$^{-1/2}$]**	**c [mg g^{-1}]**	**AdjR^2**
CS:Soot 1:2	1.22	2.61E-01	0.9852
CS:Soot 1:1	0.94	3.83E-01	0.9784
CS:Soot 2:1	0.90	5.63E-01	0.9627
CS:Soot-ox 1:2	1.72	1.67E-01	0.9834
CS:Soot-ox 1:1	1.65	8.64E-01	0.9854
CS:Soot-ox 2:1	1.62	1.34E-01	0.9710

S3. Fitting of adsorption isotherms

The experimental adsorption isotherm data was fitted to the two-parameter Langmuir and Freundlich models, and to the three-parameter Sips and Toth models (Equations S5-9, respectively) [R2-R4]:

$$q_e = q_L \frac{k_L C_e}{1 + k_L C_e} \quad \text{(S5)}$$

$$q_e = k_F C_e^{\frac{1}{n_F}} \quad \text{(S6)}$$

$$q_e = q_s \frac{(k_s C_e)^{1/n_s}}{1 + (k_s C_e)^{1/n_s}} \quad \text{(S7)}$$

$$q_e = q_T \frac{K_T C_e}{(1 + (K_T C_e)^Z)^{\frac{1}{Z}}} \quad \text{(S8)}$$

where, q_L is the maximum Langmuir adsorption capacity (mg/g), K_L is Langmuir constant (L/mg), C_e is equilibrium concentration of pollutants (mg/L); k_F is Freundlich adsorption isotherm constant which have a relation with adsorption capacity and n_F is a parameter which shows adsorbent-adsorbate affinity; q_s is the adsorption capacity at equilibrium, while k_s represents the affinity constant and n_s shows the heterogeneity of the system (with $n_s = 1$) recovering the Langmuir model; q_T is the maximum Toth adsorption capacity (mg/g), K_T (L/mg) is the Toth constant, and Z (dimensionless) is the Toth heterogeneity constant.

Table S5. Best fitting parameters for the Langmuir model.

Cr(VI) 30°C			
**Adsorbent**	q_L **[mg g⁻¹]**	k_L **[L mg⁻¹]**	**Adj**R^2
CS:Soot 1:2	58.05	4.06E-02	0.9749
CS:Soot 1:1	57.48	6.51E-02	0.9961
CS:Soot 2:1	65.80	5.86E-02	0.9838
CS:Soot-ox 1:2	61.14	3.51E-02	0.9494
CS:Soot-ox 1:1	61.33	1.62E-01	0.9067
CS:Soot-ox 2:1	75.16	9.91E-02	0.9925
**R6G 30°C**			
**Adsorbent**	q_L **[mg g⁻¹]**	k_L **[L mg⁻¹]**	**Adj**R^2
CS:Soot 1:2	72.18	2.01E-02	0.9081
CS:Soot 1:1	45.02	3.60E-02	0.9594
CS:Soot 2:1	42.70	3.12E-02	0.9455
CS:Soot-ox 1:2	47.42	1	0.6278
CS:Soot-ox 1:1	38.61	1	0.6820
CS:Soot-ox 2:1	34.11	1	0.5701

Cr(VI) 40°C			
**Adsorbent**	q_L **[mg g⁻¹]**	k_L **[L mg⁻¹]**	**Adj**R^2
CS:Soot 1:2	78.35	2.64E-02	0.9892
CS:Soot 1:1	78.60	5.29E-02	0.9959
CS:Soot 2:1	82.73	4.97E-02	0.9844
CS:Soot-ox 1:2	94.92	2.14E-02	0.9807
CS:Soot-ox 1:1	71.65	1.47E-01	0.9769
CS:Soot-ox 2:1	81.03	1.69E-01	0.9854
**R6G 40°C**			
**Adsorbent**	q_L **[mg g⁻¹]**	k_L **[L mg⁻¹]**	**Adj**R^2
CS:Soot 1:2	64.55	4.88E-02	0.8762

CS:Soot 1:1	46.11	9.66E-02	0.9533
CS:Soot 2:1	42.97	7.73E-02	0.9382
CS:Soot-ox 1:2	76.37	1.85E-02	0.9566
CS:Soot-ox 1:1	64.48	1.26E-01	0.9767
CS:Soot-ox 2:1	61.84	7.63E-02	0.9861

**Cr(VI) 50°C**			
**Adsorbent**	q_L **[mg g⁻¹]**	k_L **[L mg⁻¹]**	**Adj**R^2
CS:Soot 1:2	68.48	4.79E-02	0.9925
CS:Soot 1:1	83.44	0.032E-02	0.9948
CS:Soot 2:1	70.59	6.45E-02	0.9764
CS:Soot-ox 1:2	77.82	2.2 E-02	0.9574
CS:Soot-ox 1:1	62.70	1.63E-02	0.9736
CS:Soot-ox 2:1	71.54	2.59E-01	0.9718
**R6G 50°C**			
**Adsorbent**	q_L **[mg g⁻¹]**	k_L **[L mg⁻¹]**	**Adj**R^2
CS:Soot 1:2	69.73	0.057E-02	0.8803
CS:Soot 1:1	52.26	0.106E-02	0.9626
CS:Soot 2:1	47.88 (	9.63E-02	0.9640
CS:Soot-ox 1:2	95.61	2.82E-02	0.9662
CS:Soot-ox 1:1	72.37	1.90E-02	0.9798
CS:Soot-ox 2:1	64.56	1.20E-01	0.9798

Table S6. Best fitting parameters for the Freundlich model.

**Cr(VI) 30°C**			
**Adsorbent**	k_F **[(mg g⁻¹) (mg L⁻¹)⁻¹/ⁿ]**	n_F [-]	**Adj**R^2
CS:Soot 1:2	5.45	4.91E-01	0.9781
CS:Soot 1:1	7.24	4.52E-02	0.9604

CS:Soot 2:1	7.42	4.76E-02	0.9714
CS:Soot-ox 1:2	4.48	5.39E-02	0.8893
CS:Soot-ox 1:1	13.44	3.52E-02	0.7408
CS:Soot-ox 2:1	12.13	4.18E-02	0.9295
**R6G 30°C**			
**Adsorbent**	**k_F [(mg g^{-1}) (mg L^{-1})$^{-1/n}$]**	n_F [-]	**Adj**R^2
CS:Soot 1:2	7.56	9.29E-02	0.9536
CS:Soot 1:1	5.50	3.92 E-02	0.9879
CS:Soot 2:1	4.66	4.09E-01	0.9768
CS:Soot-ox 1:2	15.40	2.66E-02	0.8951
CS:Soot-ox 1:1	14.78	2.23E-02	0.89474
CS:Soot-ox 2:1	15.88	1.77E-01	0.8139

**Cr(VI) 40°C**			
**Adsorbent**	**k_F [(mg g^{-1}) (mg L^{-1})$^{-1/n}$]**	n_F [-]	**Adj**R^2
CS:Soot 1:2	4.10	5.99E-01	0.9614
CS:Soot 1:1	7.68	5.070E-01	0.9543
CS:Soot 2:1	7.16	5.34E-01	0.9322
CS:Soot-ox 1:2	3.96	6.34E-01	0.9651
CS:Soot-ox 1:1	14.25	3.80E-01	0.8634
CS:Soot-ox 2:1	16.72	3.81E-01	0.8900
**R6G 40°C**			
**Adsorbent**	**k_F [(mg g^{-1}) (mg L^{-1})$^{-1/n}$]**	n_F [-]	**Adj**R^2
CS:Soot 1:2	13.98	2.84E-01	0.9781
CS:Soot 1:1	10.45	2.96E-01	0.9846
CS:Soot 2:1	8.25	3.25E-01	0.9582
CS:Soot-ox 1:2	20.40	2.79E-01	0.9596
CS:Soot-ox 1:1	15.07	2.96E-01	0.9180
CS:Soot-ox 2:1	11.36	3.34E-01	0.9252

Cr(VI) 50°C			
**Adsorbent**	k_F **[(mg g^{-1}) (mg L^{-1})$^{-1/n}$]**	n_F [-]	**Adj**R^2
CS:Soot 1:2	6.54	5.02E-01	0.9583
CS:Soot 1:1	4.93	5.89E-02	0.9608
CS:Soot 2:1	9.46	4.39E-01	0.9771
CS:Soot-ox 1:2	3.31	6.30E-01	0.9196
CS:Soot-ox 1:1	13.51	3.60E-01	0.8581
CS:Soot-ox 2:1	20.14	3.16E-02	0.9776
**R6G 50°C**			
**Adsorbent**	k_F **[(mg g^{-1}) (mg L^{-1})$^{-1/n}$]**	n_F [-]	**Adj**R^2
CS:Soot 1:2	16.06	2.77E-02	0.9817
CS:Soot 1:1	12.69	2.83E-02	0.9794
CS:Soot 2:1	10.92	2.93E-02	0.9436
CS:Soot-ox 1:2	32.20	2.34E-02	0.9597
CS:Soot-ox 1:1	20.17	2.65E-02	0.8672
CS:Soot-ox 2:1	14.95	2.97E-01	0.9392

Table S7. Best fitting parameters for the Sips model.

Cr(VI) 30°C				
**Adsorbent**	q_s **[mg g^{-1}]**	n_s [-]	k_s **[L mg^{-1}]**	**Adj**R^2
CS:Soot 1:2	58.05	2.81E-01	1.14E-02	0.9708
CS:Soot 1:1	57.48	5.27	3.42E-01	0.9955
CS:Soot 2:1	65.80	1.56	9.17	0.9812
CS:Soot-ox 1:2	61.14	2.42E-01	97.6 E-03	0.9410
CS:Soot-ox 1:1	61.35	5.91E-02	9.63E-3	0.8911
CS:Soot-ox 2:1	75.16	1.03	1.02E-01	0.9913
**R6G 30°C**				
**Adsorbent**	q_s **[mg g^{-1}]**	n_s [-]	k_s **[L mg^{-1}]**	**Adj**R^2
CS:Soot 1:2	72.18	3.955E-02	7.98228E-4	0.8897

CS:Soot 1:1	45.02	2.03E-03	7.30011E-5	0.9512
CS:Soot 2:1	42.70	2.49 E-03	7.78E-5	0.9346
CS:Soot-ox 1:2	46.03	2.77E-01	1.05	0.5923
CS:Soot-ox 1:1	37.49	6.005 E-01	1.47E-01	0.6567
CS:Soot-ox 2:1	32.88	1.89 E-01	9.67E-01	0.6409

Cr(VI) 40°C				
**Adsorbent**	q_s [mg g^{-1}]	n_s [-]	k_s [L mg^{-1}]	AdjR^2
CS:Soot 1:2	78.35	1.66E-01	4.39E-03	0.9874
CS:Soot 1:1	78.60	2.3E-01	1.22E-02	0.9953
CS:Soot 2:1	82.73	7.83	3.90E-01	0.9820
CS:Soot-ox 1:2	94.92	1.31E-01	2.83	0.9775
CS:Soot-ox 1:1	71.65	495.74	73.203	0.9730
CS:Soot-ox 2:1	81.03	2.87	49.79	0.9830
**R6G 40°C**				
**Adsorbent**	q_s [mg g^{-1}]	n_s [-]	k_s [L mg^{-1}]	AdjR^2
CS:Soot 1:2	64.55	1.25E-01	2.57E-01	0.9328
CS:Soot 1:1	46.11	3.72E-02	3.84E-01	0.9440
CS:Soot 2:1	42.97	1.20E-02	9.28E-4	0.9259
CS:Soot-ox 1:2	76.37	3.32E-12	6.27E-13	0.9483
CS:Soot-ox 1:1	64.48	5.57E-7	7053.5	0.9720
CS:Soot-ox 2:1	61.84	3.99E-01	3.05E-02	0.9832

Cr(VI) 50°C				
**Adsorbent**	q_s [mg g^{-1}]	n_s [-]	k_s [L mg^{-1}]	AdjR^2
CS:Soot 1:2	68.48	4.53E-6	2.17E-7	0.9913
CS:Soot 1:1	83.44	1.31	4.16E-02	0.9932
CS:Soot 2:1	70.59	5.20E-02	3.36E-03	0.9724

CS:Soot-ox 1:2	77.82	1.35	2.98E-02	0.9503
CS:Soot-ox 1:1	62.70	2.81E-01	4.59E-02	0.9692
CS:Soot-ox 2:1	71.59	1.13E7	2938168.06	0.9671
**R6G 50°C**				
**Adsorbent**	q_s **[mg g^{-1}]**	n_s **[-]**	k_s **[L mg^{-1}]**	**Adj**R^2
CS:Soot 1:2	69.73	4.18E7	23513.8	0.8564
CS:Soot 1:1	52.26	9.05E-01	0.097	0.9552
CS:Soot 2:1	47.88	2.47E-6	2.18E-7	0.9568
CS:Soot-ox 1:2	95.61	3.44	9.71E-02	0.9595
CS:Soot-ox 1:1	72.37	4.59E-02	8.76E-02	0.9757
CS:Soot-ox 2:1	64.56	6.99E8	8.45E7	0.9758

Table S8. Best fitting parameters for the Toth model.

**Cr(VI) 30°C**				
**Adsorbent**	q_T **[mg g^{-1}]**	k_T **[L mg^{-1}]**	**Z [-]**	**Adj**R^2
CS:Soot 1:2	37.39	6.79E-02	8.47E-01	0.9928
CS:Soot 1:1	57.90	6.44E-02	1.002	0.9955
CS:Soot 2:1	38.02	1.17E-01	8.83E-01	0.9904
CS:Soot-ox 1:2	47.27	3.76E-02	8.94E-01	0.9817
CS:Soot-ox 1:1	89.94	7.64E-02	1.15	0.9569
CS:Soot-ox 2:1	102.87	6.84E-02	1.12	0.9953
**R6G 30°C**				
**Adsorbent**	q_T **[mg g^{-1}]**	k_T **[L mg^{-1}]**	**Z [-]**	**Adj**R^2
CS:Soot 1:2	52.99	4.59E-02	9.27E-01	0.9992
CS:Soot 1:1	29.61	7.76E-02	8.81E-01	0.9963
CS:Soot 2:1	33.86	6.41E-029	9.36E-01	0.9976
CS:Soot-ox 1:2	38.96	1.53E-01	8.78E-01	0.9990
CS:Soot-ox 1:1	18.46	3.36E-01	8.02E-01	0.9962

CS:Soot-ox 2:1	19.924	4.92E-01	8.65E-01	0.98467

Cr(VI) 40°C				
**Adsorbent**	**q_T [mg g^{-1}]**	**k_T [L mg^{-1}]**	**Z [-]**	**AdjR^2**
CS:Soot 1:2	62.68	3.90E-02	9.72E-01	0.9989
CS:Soot 1:1	75.52	5.13E-02	9.82 E-01	0.9962
CS:Soot 2:1	103.34	4.02E-02	1.18	0.9824
CS:Soot-ox 1:2	71.89	3.68E-02	9.52E-01	0.9971
CS:Soot-ox 1:1	109.00	9.34E-02	1.16	0.9807
CS:Soot-ox 2:1	117.80	1.14E-01	1.13	0.9883
**R6G 40°C**				
**Adsorbent**	**q_T [mg g^{-1}]**	**k_T [L mg^{-1}]**	**Z [-]**	**AdjR^2**
CS:Soot 1:2	54.94	3.34E-02	9.44E-01	0.9989
CS:Soot 1:1	45.69	8.29E-02	9.70E-01	0.9986
CS:Soot 2:1	38.74	8.74E-02	9.55E-01	0.9971
CS:Soot-ox 1:2	75.45	1.17E-01	9.70E-01	0.9898
CS:Soot-ox 1:1	48.22	1.99E-01	9.29E-01	0.9854
CS:Soot-ox 2:1	51.22	9.12E-02	9.44E-01	0.9790

Cr(VI) 50°C				
**Adsorbent**	**q_T [mg g^{-1}]**	**k_T [L mg^{-1}]**	**Z [-]**	**AdjR^2**
CS:Soot 1:2	73.13	4.36E-02	1.02	0.9926
CS:Soot 1:1	73.09	3.88E-02	9.70E-01	0.9986
CS:Soot 2:1	69.31	6E-02	9.75E-01	0.9857
CS:Soot-ox 1:2	62.08	2.39E-02	8.83E-01	0.9934
CS:Soot-ox 1:1	90.29	1.07E-01	1.13	0.9749
CS:Soot-ox 2:1	103.22	1.08E-01	1.09	0.9959
**R6G 50°C**				
**Adsorbent**	**q_T [mg g^{-1}]**	**k_T [L mg^{-1}]**	**Z [-]**	**AdjR^2**

CS:Soot 1:2	66.44	8.31E-02	9.77E-01	0.9980
CS:Soot 1:1	49.77	9.71E-01	9.70E-01	0.9898
CS:Soot 2:1	46.95	8.46E-02	9.79E-01	0.9795
CS:Soot-ox 1:2	92.08	2.02E-01	9.78E-01	0.9820
CS:Soot-ox 1:1	68.12	2.07 E-01	9.81 E-01	0.98384
CS:Soot-ox 2:1	49.73	1.29	9.23	0.9703

S4. Equilibrium adsorption capacity for the effect of adsorbent dosage

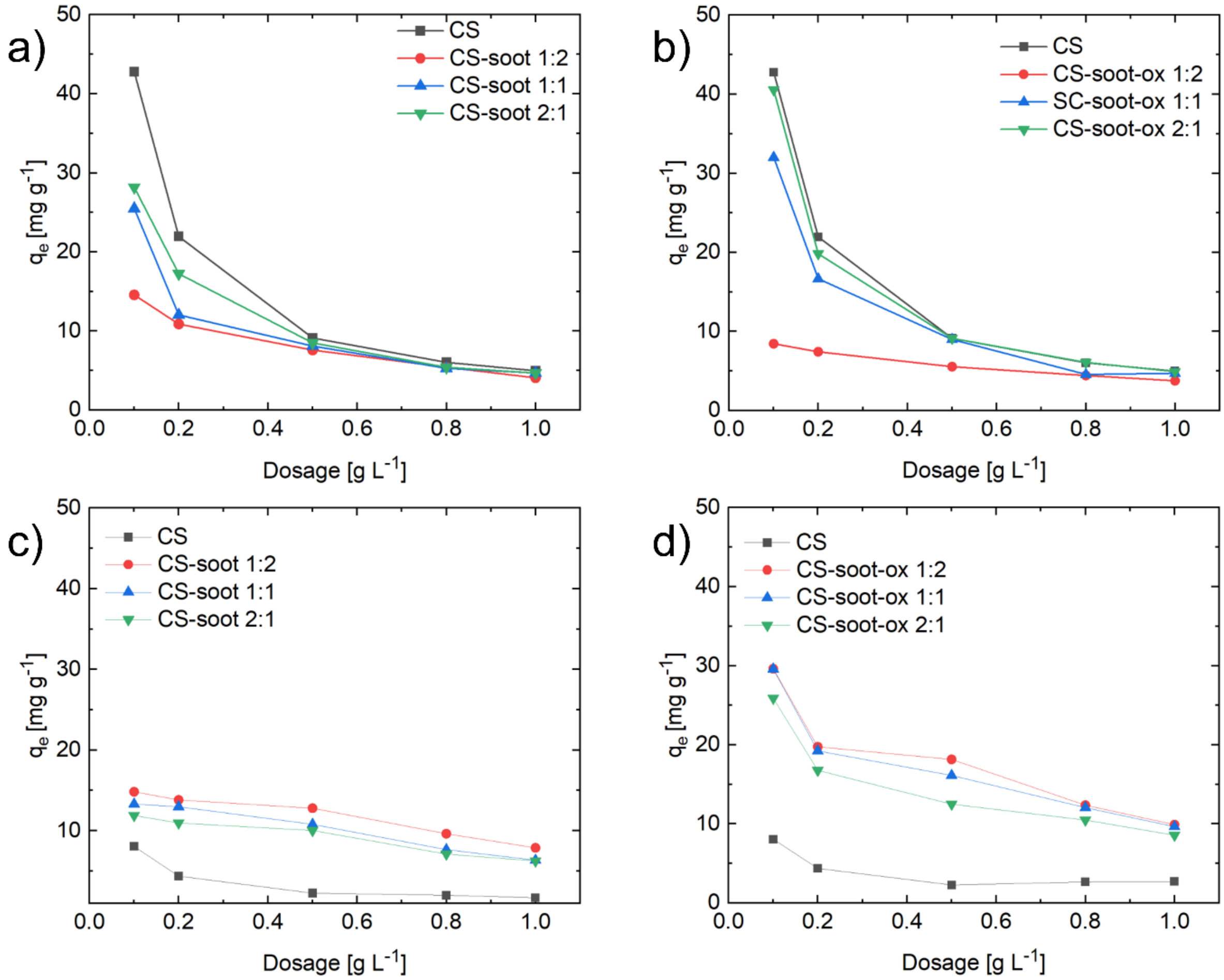

Figure S2. Effect of adsorbent dosage on the removal of a,b) Cr(VI) and c,d) R6G (T=30°C, contact time 150 minutes, pH = 5 for Cr(VI) and pH = 9 for R6G) with a,c) CS:Soot and b,d) CS:Soot-ox aerogels at different composition. Dashed lines are guide for the eye.

References

[R1] Wang, J.; Guo, X. Adsorption kinetic models: Physical meanings, applications, and solving methods. J. Hazard. Mater. 2020, 390, No. 122156.

[R2] Al-Ghouti, M. A., & Da'ana, D. A. (2020). Guidelines for the use and interpretation of adsorption isotherm models: A review. *Journal of hazardous materials*, *393*, 122383.

[R3] Wang, J., & Guo, X. (2020). Adsorption isotherm models: Classification, physical meaning, application and solving method. *Chemosphere*, *258*, 127279.

[R4] Kyzas, G. Z., Siafaka, P. I., Pavlidou, E. G., Chrissafis, K. J., & Bikiaris, D. N. (2015). Synthesis and adsorption application of succinyl-grafted chitosan for the simultaneous removal of zinc and cationic dye from binary hazardous mixtures. *Chemical Engineering Journal*, *259*, 438-448.
